# Host CLIC4 expression in the tumor microenvironment is essential for breast cancer metastatic competence

**DOI:** 10.1371/journal.pgen.1010271

**Published:** 2022-06-21

**Authors:** Vanesa C. Sanchez, Howard H. Yang, Alayna Craig-Lucas, Wendy Dubois, Brandi L. Carofino, Justin Lack, Jennifer E. Dwyer, R. Mark Simpson, Christophe Cataisson, Max P. Lee, Ji Luo, Kent W. Hunter, Stuart H. Yuspa

**Affiliations:** 1 Laboratory of Cancer Biology and Genetics, Center for Cancer Research, National Cancer Center, National Institutes of Health, Bethesda, Maryland, United States of America; 2 NIAID Collaborative Bioinformatics Resource (NCBR), National Institute of Allergy and Infectious Disease, National Institutes of Health, Bethesda, Maryland, United States of America; 3 Advanced Biomedical Computational Science, Frederick National Laboratory for Cancer Research, Leidos Biomedical Research, Inc., Frederick, Maryland, United States of America; University of Milan, ITALY

## Abstract

The TGF-β-regulated Chloride Intracellular Channel 4 (CLIC4) is an essential participant in the formation of breast cancer stroma. Here, we used data available from the TCGA and METABRIC datasets to show that *CLIC4* expression was higher in breast cancers from younger women and those with early-stage metastatic disease. Elevated *CLIC4* predicted poor outcome in breast cancer patients and was linked to the TGF-β pathway. However, these associations did not reveal the underlying biological contribution of CLIC4 to breast cancer progression. Constitutive ablation of host *Clic4* in two murine metastatic breast cancer models nearly eliminated lung metastases without reducing primary tumor weight, while tumor cells ablated of *Clic4* retained metastatic capability in wildtype hosts. Thus, CLIC4 was required for host metastatic competence. Pre- and post-metastatic proteomic analysis identified circulating pro-metastatic soluble factors that differed in tumor-bearing CLIC4-deficient and wildtype hosts. Vascular abnormalities and necrosis increased in primary tumors from CLIC4-deficient hosts. Transcriptional profiles of both primary tumors and pre-metastatic lungs of tumor-bearing CLIC4-deficient hosts were consistent with a microenvironment where inflammatory pathways were elevated. Altogether, CLIC4 expression in human breast cancers may serve as a prognostic biomarker; therapeutic targeting of CLIC4 could reduce primary tumor viability and host metastatic competence.

## Author summary

CLIC4, a member of a highly conserved CLIC gene family, contributes to the pathogenesis of several human diseases. We show that elevated *CLIC4* expression in human breast cancers predicts early invasion and poor outcome in women. Further, in murine models of breast cancer, CLIC4 in the host microenvironment is required for lung metastasis. In the absence of host CLIC4, the microenvironment of the primary tumor and the pre-metastatic lungs is unfavorable for tumor viability and lung colonization. These findings introduce CLIC4 as a potential therapeutic target against a host protein to prevent metastatic spread in breast cancer.

## Introduction

Chloride Intracellular Channel 4 (CLIC4) belongs to a family of highly conserved metamorphic proteins of the glutathione-S-transferase superfamily. In isolated model systems, CLIC proteins participate in multiple cellular functions [1–5], and CLIC4 has been implicated in the pathogenesis of several human diseases [6–8]. In human cancers, CLIC4 protein expression varies, being characteristically lost in progression of cancer cells such as those of the esophagus and colon [9] but highly expressed in other cancer cells such as those of the ovary and pancreas [10,11]. In almost all cancers studied, CLIC4 is highly expressed in the cancer stroma and tightly linked to cancer progression and the formation of a myofibroblastic cancer stroma [9,12,13]. Putative functions of CLIC4 that could impact its role in cancer stroma include neoangiogenesis [14,15], endothelial morphogenesis and tubulogenesis [16,17], wound healing [18], redox regulation [19], and cell adhesion [20]. At the cellular level, CLIC4 also participates in enhancing TGF-β activity by preventing dephosphorylation of phospho-SMADs in the nucleus [21] and inducing a dominant-negative splice variant of inhibitory SMAD7 [22]. In return, TGF-β induces expression and nuclear translocation of CLIC4 [21].

Recently, CLIC4 has become a molecule of interest in breast cancer [13,23], where it is expressed in stromal cells [12]. *CLIC4* expression is highly responsive to TGF-β in primary human mammary fibroblasts [12], and human breast cancer cells release TGF-β that induces the conversion of fibroblasts to myofibroblasts through a CLIC4-dependent pathway [13]. Tumors evolving from xenografts of human breast cancer cells are substantially larger if co-transplanted with fibroblasts genetically altered to express high levels of CLIC4 [9]. Interstitial fluid, extracted through drains from mastectomy sites of patients with highly aggressive breast cancers, strongly induce CLIC4 in cultured human breast cancer cells relative to interstitial fluid from mastectomy patients with low grade lesions [23]. This high-grade fluid also causes myofibroblast conversion of mammary fibroblasts. Thus, crosstalk between the tumor and host stroma involves changes in CLIC4 expression. CLIC4 is also detected in human plasma [6] and may circulate as a free molecule or as cargo in extracellular vesicles [24].

In this current study, we established a relationship between elevated CLIC4 expression in multiple breast cancer data sets and the progressive clinical course of human breast cancer. However, bulk tumor measurements of a biomarker do not establish if there is a disease contribution or reveal a mechanism. Therefore, we genetically manipulated *Clic4* in the host or the tumor cells in syngeneic immunocompetent murine breast cancer models with reproducible lung metastases to clarify whether CLIC4 in the host and/or in the tumor cells is required for the development and progression of breast cancer. We discovered that host CLIC4 is required for breast cancer metastasis and identified CLIC4 functions that may contribute to metastatic competence.

## Results

### *CLIC4* expression in human breast cancers correlates with poor outcome

**Fig 1:**
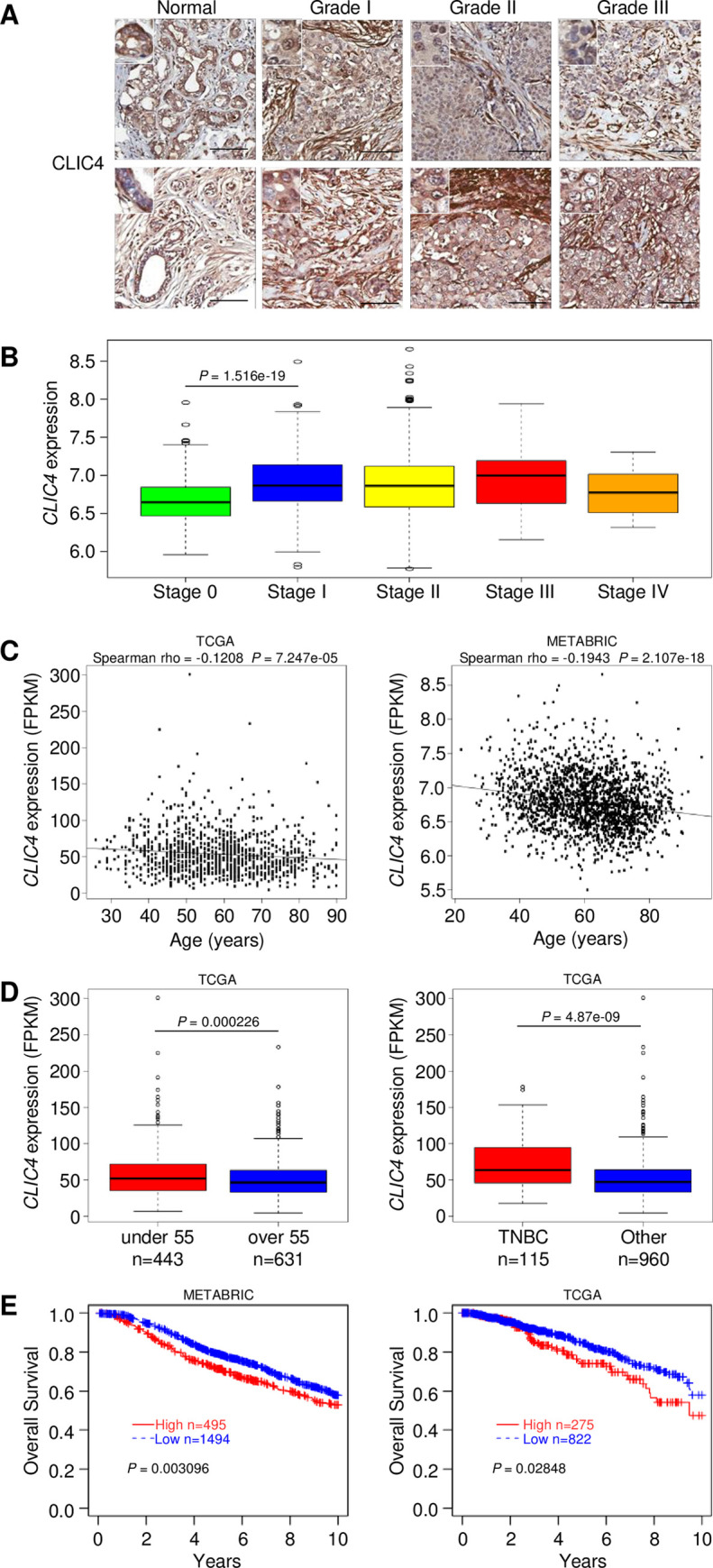
CLIC4 is expressed in human breast cancers and varies with patient age and survival probability. (*A*) Representative distribution of CLIC4 expression in human breast cancer tissue arrays by IHC. Scale bar, 100 μm. Each panel illustrates a different tumor. (*B*) Box plots of *CLIC4* expression in breast tumors separated by stage from the METABRIC database. (*C*) Correlation analysis of *CLIC4* mRNA expression and patient age in human breast cancer patients (FPKM = fragments per kilobase of exon per million reads mapped). Left panel, TCGA-BRCA (n = 1075). Right panel, METABRIC (n = 1992). (*D*) Comparison of *CLIC4* expression in cancers from women with breast cancer below and above 55 years of age (left panel, n = 1074). Comparison of *CLIC4* expression in cancers from women with triple-negative breast cancer (TNBC) and other breast cancer types (right panel, n = 1075). Source data from TCGA-BRCA (FPKM = fragments per kilobase of exon per million reads mapped). (*E*) METABRIC and TCGA-BRCA breast cancer datasets, with 75th percentile as the cut-off and 10-year censored data, show the high *CLIC4* group had poor survival compared to the low *CLIC4* expression group compared by the Kaplan-Meier curves.

Immunostaining of human breast and breast cancer tissue arrays revealed CLIC4 staining primarily in the acini and ducts of normal breast tissue that became more diffuse and intense, particularly in the stromal compartments of breast cancers (Fig 1A). To assess whether *CLIC4* expression in tumor tissue correlated with any clinical data in breast cancer patients, we analyzed data from the Molecular Taxonomy of Breast Cancer International Consortium (METABRIC) study [25] and The Cancer Genome Atlas (TCGA) database [26]. METABRIC data revealed that *CLIC4* expression in breast cancers significantly increased in patients progressing from Stage 0 to Stage I and remained elevated compared to those with *in situ* lesions (Fig 1B). *CLIC4* expression was higher in cancers of younger patients relative to that of cancers from older patients (Fig 1C). TCGA confirmed higher *CLIC4* expression in tumors from women under 55 years of age and in triple-negative breast cancers (TNBC) compared to other pathological types (Fig 1D). These correlations, potentially skewed by the highly significant association with TNBC, suggested that higher *CLIC4* expression in breast cancers was associated with more aggressive disease and poor outcome. Kaplan-Meier curves **(**Figs 1E and S1A**)** indicated that survival decreased with high *CLIC4* expression using 10-year censored data from both the METABRIC and TCGA-BRCA datasets. This suggested that the elevation of CLIC4 had significant prognostic implications mainly within 10 years after the diagnosis for breast cancer patients. Since consistent CLIC4 structural genomic alterations were not detected in human cancers in our previous study [27], we reasoned that elevated CLIC4 in progressive breast cancer was likely related to TGF-β pathway expression, a major regulator of CLIC4 expression [21] and an important influence on breast cancer outcome [28]. To evaluate possible correlations between TGF-β family members and CLIC4, we analyzed two protein datasets in the NCI Proteomic Data Commons, the TCGA Breast Cancer Proteome [29] with n = 108 cases and the Prospective Breast BI Proteome [30] with n = 125 cases. After combining the two protein datasets, we calculated the Spearman correlations between CLIC4 and eight TGF-β pathway protein members. Six out of the eight proteins examined showed a significant correlation with Spearman rho values greater than 0.17 and p-values less than 8e-3. The four most significant correlated proteins TGFB1, TGFB1I1, TGFB2, and TGFBI with Spearman rho values greater than 0.37 and p-values less than 5e-10 are shown as scatter plots (S1B–S1E Fig). These data implicate CLIC4 as a downstream effector of TGF-β signaling in breast cancer.

### Metastatic breast cancer models reveal host but not tumor cell dependence on CLIC4

**Fig 2:**
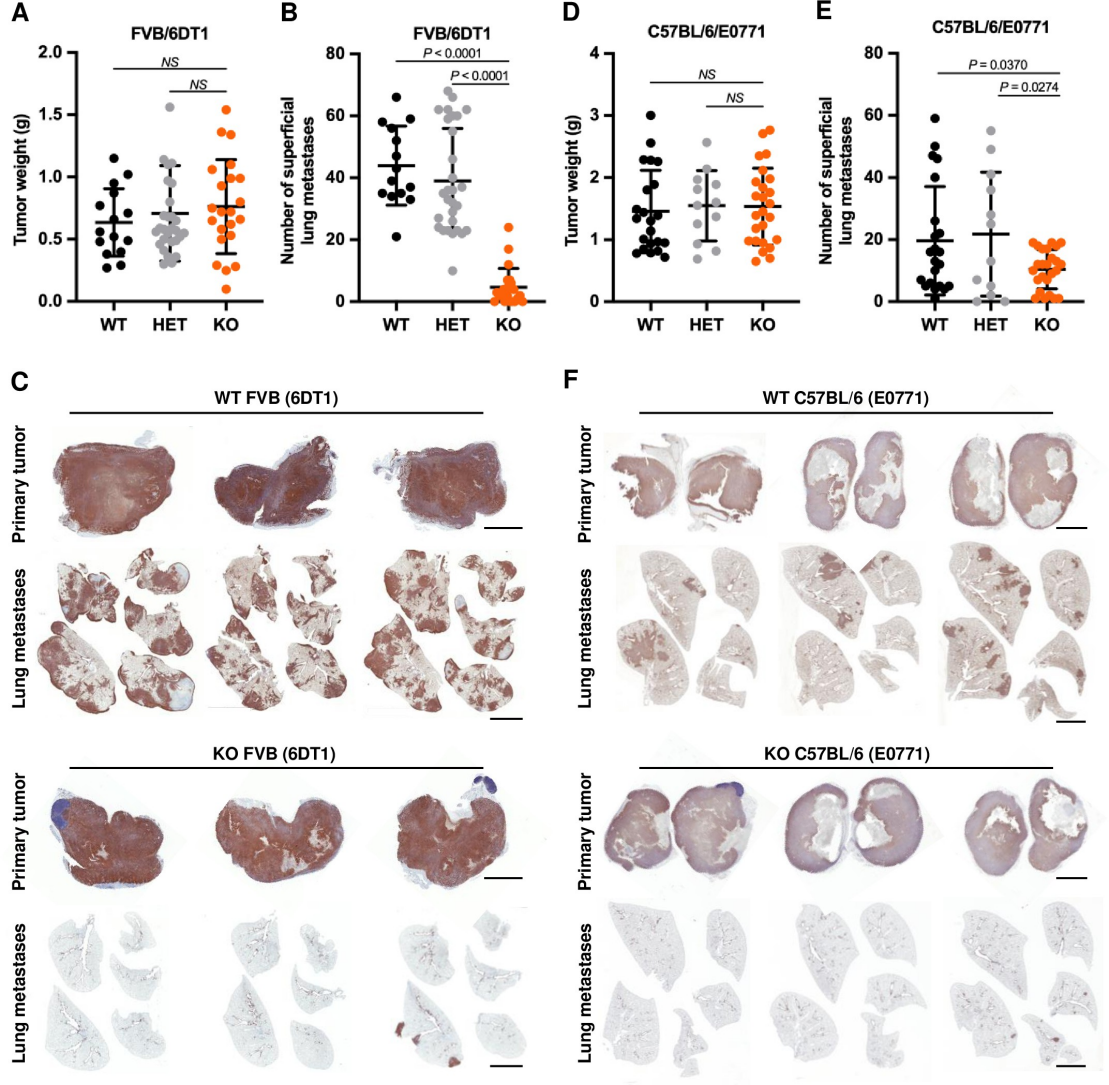
*Clic4* host deficiency prevents lung metastases in syngeneic breast cancer models. (*A-C*) 1×10^5^ 6DT1 cells were implanted into the fourth left mammary fat pad of *Clic4* wildtype (WT), n = 15, heterozygous (HET), n = 29, and knockout (KO), n = 22, FVB female mice. Primary tumors were resected and weighed (g = grams) (*A*), and superficial lung metastases quantified (*B*) after 28 days. (*C*) Immunohistochemistry for CLIC4 expression in 6DT1 primary tumors and superficial lung metastases from individual mice at 28 days post-implantation. (*D-F*) Primary tumor weight (g = grams) (*D*), number of lung metastases (*E*), and CLIC4 immunohistochemistry (*F)* 28 days after implanting 2.5×10^5^ E0771 cells into *Clic4* wildtype (WT), n = 22, heterozygous (HET), n = 12, and knockout (KO), n = 25, C57BL/6 female mice. Values represent the mean ± s.d. P values were obtained after ANOVA followed by unpaired t test. Scale bar, 4 mm.

Bulk tumor data imply a connection to disease but do not reveal mechanism. Therefore, we turned to animal models to address the contribution of CLIC4 in the development of primary and metastatic breast cancer. For these studies, we used the 6DT1 and E0771 models, both previously characterized syngeneic breast cancer models with lung metastatic tropism. Both models form predictable primary tumors and lung metastases following implantation of primary tumor cells in the mammary fat pad of female mice [31–33]. FVB/6DT1 tumor cells were derived from a mammary tumor arising from a MMTV-Myc transgenic mouse and C57BL/6/E0771 tumor cells were derived from a spontaneous adenocarcinoma in the mammary gland of a C57BL/6 mouse [33]. Both cell lines express CLIC4 (S2A Fig). *Clic4* knockout (KO) mice are fertile, develop normally, and show no constitutive health effects; evidence of diminished wound healing and impaired neoangiogenesis are detected only when challenged [14,18,21]. Breast cancer cells were injected into the 4^th^ left mammary fat pad of *Clic4* wildtype (WT) or KO female mice (day 0). In the standard 6DT1 model, primary tumors grew progressively, and multiple lung metastases in all WT mice were detected starting after 14 days and peaking at 28 days, after which sacrifice was required because of primary tumor size (S2B Fig) [33,34]. H and E staining confirmed the progressive increase in size of the primary tumor and the extent of metastatic spread (S2C Fig, left). Histological analysis showed high cell density of uniformly round epithelial cancer cells in both primary and metastatic compartments (S2C Fig, right). Primary tumors and metastases expressed CLIC4 in the epithelial and stromal compartments (S2D Fig). The model was then tested in hosts of different genotypes. The absence of host CLIC4 did not affect primary tumor weight, but nearly eliminated the number of lung metastases at 28 days post-implantation of 6DT1 tumor cells (Fig 2A and 2B). Heterozygous *Clic4* (HET) mice followed the trends observed for WT mice. Both primary tumors and any metastases that developed expressed CLIC4 in both WT and KO host genotypes (Fig 2C) as well as background lung tissue in WT but not KO mice. This unexpected result on metastatic competence was confirmed using an independent genetic model of C57BL/6-derived E0771 breast cancer cells injected into *Clic4* WT, HET, and KO C57BL/6 female mice (Fig 2D–2F). Since the mean number of metastases was higher in the FVB/6DT1 model compared to the C57BL/6/E0771, the former model was used for additional studies.

### Absence of CLIC4 alters the “seed and soil” in both the primary tumor and the lungs

**Fig 3:**
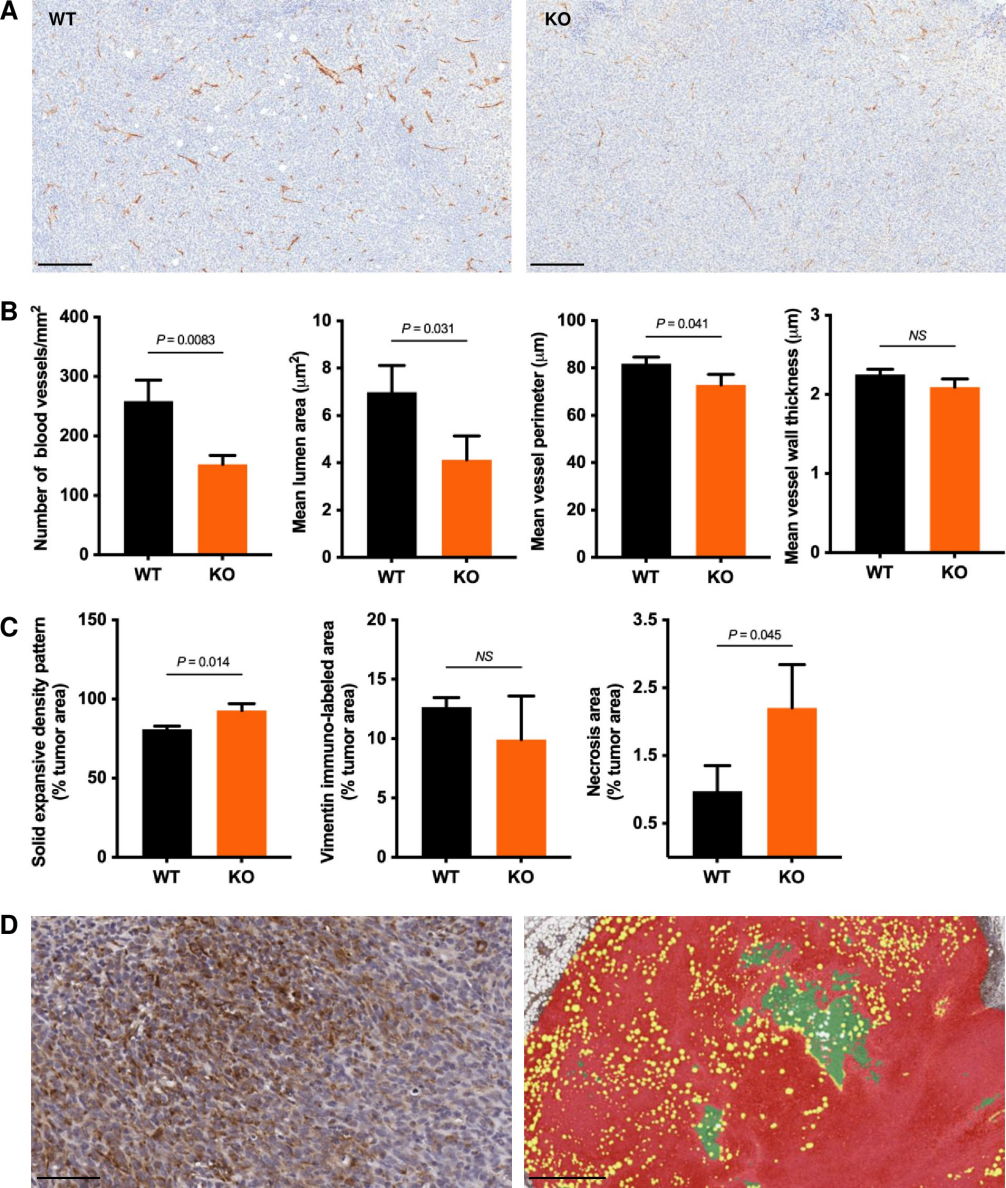
*Clic4* host deficiency impairs neovascularization of the primary tumor. (*A*) Representative CD31 immunostaining in primary tumors from *Clic4* wildtype (WT) and knockout (KO) tumor-bearing mice at 28 days post-implantation. Scale bar, 200 μm. (*B*) Quantification of tumor vascularization in primary tumors from *Clic4* wildtype (WT) and knockout (KO) tumor-bearing mice at 28 days post-implantation (n = 3 per group). Values represent the mean ± s.d. from whole tissue sections analyzed by Image Scope. P values were obtained after unpaired t test. (*C*) Quantification of solid tumor expansive density area, vimentin immuno-labeled area, and necrosis area in primary tumors from *Clic4* wildtype (WT) and knockout (KO) tumor-bearing mice at 28 days post-implantation (n = 3 per group). Values represent the mean ± s.d. from whole tissue sections analyzed by HALO imaging software. P values were obtained after unpaired t test. (*D*) Representative vimentin immunostaining (left) and pseudo-colored analysis mask (right) showing the segmentation of tumor (red), necrosis (green), and intervening loose areolar and adipose connective tissue areas (yellow) in primary tumors that were used for the quantification shown in (*C*). Scale bars, 50 μm (left) and 500 μm (right).

**Fig 4:**
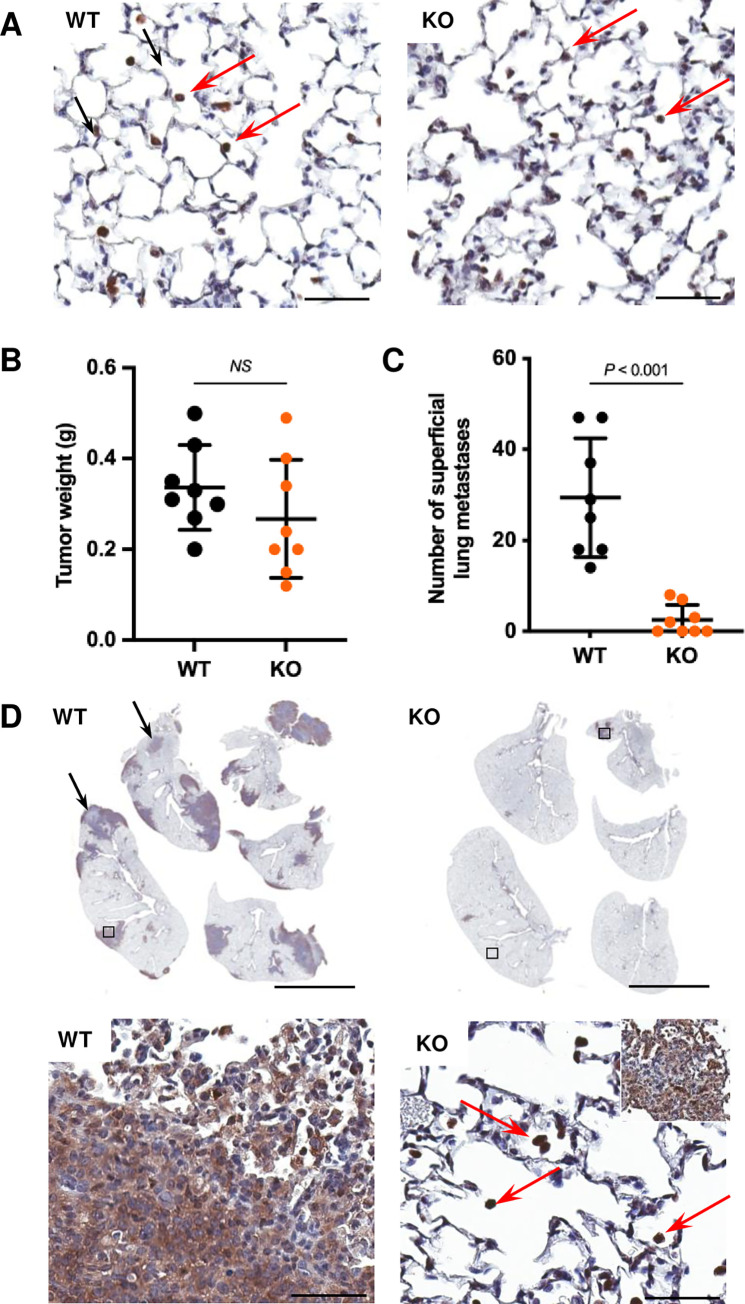
Host *Clic4* is required to support lung colonization of tail vein-injected tumor cells. Fourteen days after implantation of 1×10^5^ 6DT1 cells into the fourth left mammary fat pad of *Clic4* wildtype (WT), n = 8, and knockout (KO), n = 8, FVB females, 1×10^5^ GFP-labeled 6DT1 cells were injected via tail vein. (*A*) GFP immunostaining detects individual tail vein-injected GFP-labeled 6DT1 cells (red arrows) in lungs after 48 hours. Scale bar, 50 μm. (*B-C*) Primary tumor weight (g = grams) (*B*) and number of superficial lung metastases (*C*) 14 days post-tail vein injection. Values are means ± s.d., P values were determined by unpaired t test. (*D*) GFP-positive metastases, mixed GFP-positive and -negative metastases (black arrows), or residual single cells (red arrows) in lungs 14 days after tail vein injection of GFP-labeled 6DT1 cells. Scale bars, 5 mm (top) and 50 μm (bottom). Insert in bottom right panel, micro metastasis.

A tenet of the metastatic process states that the microenvironment of the primary tumor and the distant sites (the “seed and soil”) must be receptive to both departing and arriving cells [35,36]. Host CLIC4 could be required at multiple steps of the metastatic process either at the primary tumor or distant sites leading to lung metastases. CLIC4 is highly expressed in endothelial cells [16]. CD31 staining indicated a reduction in the number and size of blood vessels in primary tumors growing in *Clic4* KO hosts, although the thickness of vessel walls was not altered by genotype (Fig 3A and 3B). Of note, however, primary tumor weight was not affected (Fig 2A), nor was primary tumor cell density diminished by growth in *Clic4* KO hosts (Fig 3C). However, the number of necrotic foci increased in tumors from CLIC4-deficient hosts (Fig 3C and 3D) as a potential consequence of the vascular changes. Epithelial-to-mesenchymal transition (EMT) in primary breast tumors is associated with higher metastatic potential [37]. Using vimentin staining to map EMT in primary tumors, no differences could be quantified among the host genotypes (Fig 3C and 3D). To test the receptiveness of *Clic4* KO lungs to circulating tumor cells, GFP-labeled 6DT1 cells were injected into the tail vein. Preliminary studies indicated that lung tumor development in both genotypes was very poor after tail vein injection in the absence of pre-conditioning with a primary tumor as reported for other models [36,38]. Therefore, parental GFP-negative 6DT1 cells were implanted in the 4^th^ left mammary fat pad to pre-condition the lungs for 14 days followed by tail vein injection. After 14 additional days, the number of GFP-positive lung tumors were quantified (Fig 4). GFP-positive tumor cells were detected in the lungs of both WT and *Clic4* KO mice 2 days after tail vein injection (Fig 4A). Parental primary tumors grew to the same weight in both genotypes (Fig 4B). Although large GFP-positive metastases were detected in wildtype mice 14 days after tail vein injection, only a few small GFP-positive metastases developed in the *Clic4* KO lungs (Fig 4C and 4D) although individual GFP-positive injected tumor cells persisted in both genotypes. While large GFP-positive metastases from injected cells dominated the WT lungs, small isolated or integrated GFP-negative metastases were also present in WT lungs (Fig 4D, black arrows) that must have spread from the primary tumor in WT mice. These results suggested that lungs deficient in CLIC4 are poor “soil” for incoming tumor cells because they lack a supportive microenvironment and/or are insensitive to communications from the primary tumor or other cell types to form a receptive premetastatic niche. Alternatively, messages from the primary tumor growing in *Clic4* KO mice may be ineffective for conditioning the distant metastatic bed.

### CLIC4 is not required in tumor cells for growth or metastasis

**Fig 5:**
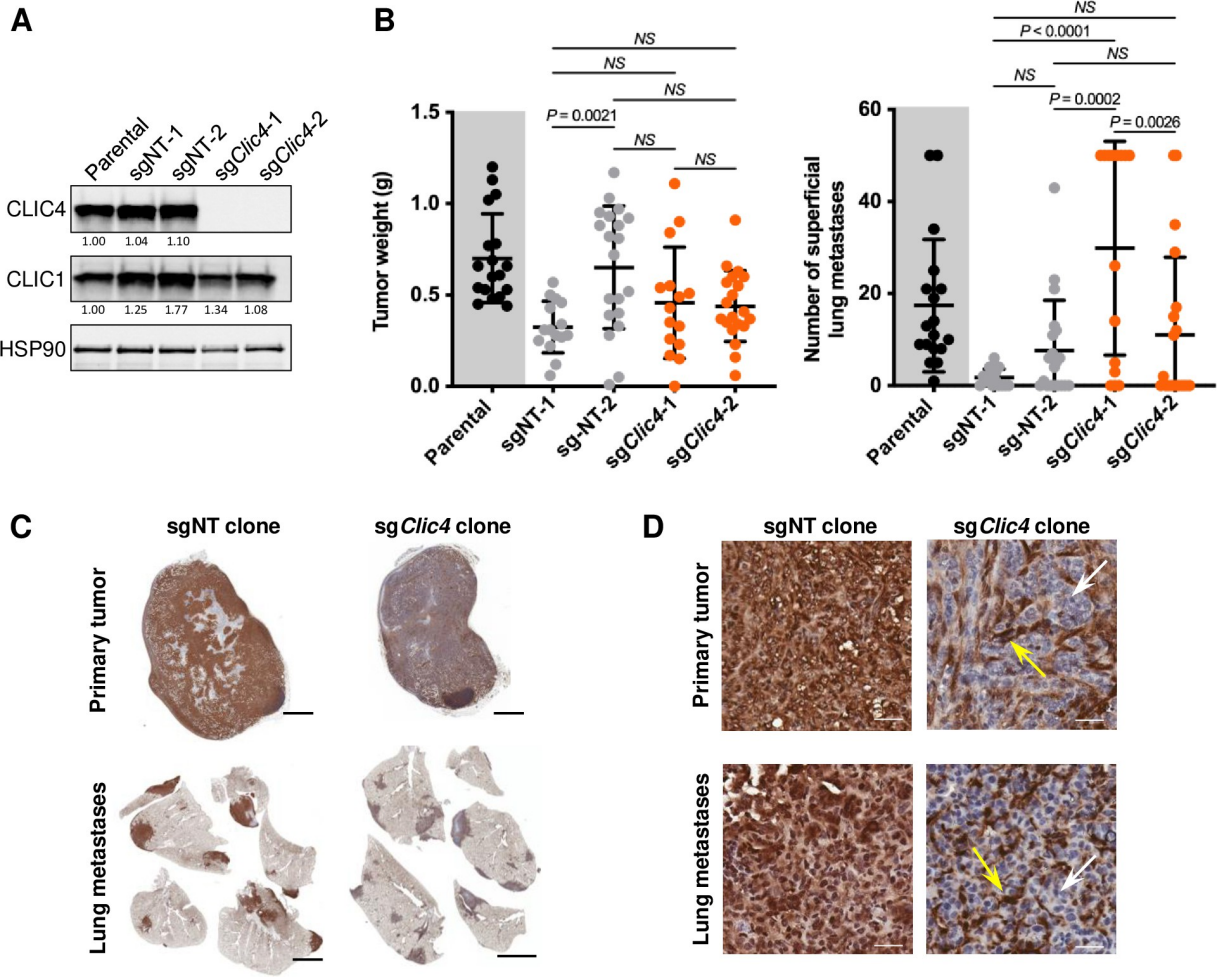
CLIC4 is not required for growth or metastatic spread of tumor cells. (*A*) Deletion of *Clic4* in 6DT1 tumor cells (Parental) by CRISPR/Cas9 was confirmed by immunoblot. Two independent *Clic4* wildtype (sgNT) and *Clic4* knockout (sg*Clic4*) clones were selected. Densitometry-based quantification is shown below each protein band, normalized to the loading control (HSP90). NT = non-targeting. (*B*) 1×10^5^ sgNT or sg*Clic4* 6DT1 cells were implanted into the fourth left mammary fat pad of *Clic4* wildtype FVB females. Primary tumor weight (g = grams) and number of superficial lung metastases were evaluated 28 days post-implantation of the clonal sublines sgNT-1 (n = 15), sgNT-2 (n = 20), sg*Clic4*-1 (n = 15), and sg*Clic4*-2 (n = 20). Parental cell-derived tumors (n = 18; gray background) are shown for reference. P values were obtained using one-way ANOVA with comparisons between all clonal lines and the Sidak correction for multiple comparisons. (*C-D*) Representative immunohistochemistry showing the tissue distribution of CLIC4 expression in primary tumor and lung metastases from tumor-bearing mice at 28 days post-implantation of 1×10^5^ sgNT or sg*Clic4* 6DT1 cells. Scale bar, 2 mm (*C*) and 30 μm (*D*). Yellow arrows, tumor stromal compartment. White arrows, tumor epithelial compartment.

The foregoing data showed that host CLIC4 was required for lung metastatic competence in these breast cancer models. To address the requirement for CLIC4 in tumor cells, CRISPR/Cas9 was used to delete *Clic4* from 6DT1 cells. Multiple clones were selected following transduction with either a non-targeting guide RNA (sgNT) or a guide RNA targeting *Clic4* (sg*Clic4*) (Table 1), and clones that lacked CLIC4 were selected (Fig 5A). The specificity of the knockout was confirmed by the preservation of the closely related CLIC1 protein expression at levels slightly above parental cells in all the clones. CLIC1 and CLIC4 are the only CLIC paralogues expressed in 6DT1 cells and derivative clones. *In vivo*, WT mice implanted with either sgNT or sg*Clic4* clones developed primary tumors and multiple lung metastases by 28 days. While the sgNT-1 clone formed smaller tumors and the number of metastases varied both within the sgNT/sg*Clic4* groups and between individual clones, this is not surprising given that subclones may have differences in tumor-forming potential and timeline. Nevertheless, the deletion of CLIC4 did not interfere with the ability of tumor cells to form a primary tumor and spread metastatically in WT hosts (Fig 5B). Immunohistochemical analysis showed both primary tumor and metastases from sg*Clic4* tumor-bearing mice lacked CLIC4 in tumor cells while host stromal cells were positive (Fig 5C and 5D). Therefore, CLIC4-deficient cells are capable of metastasis and host alterations underpin the reduced metastasis observed in *Clic4* KO mice.

**Table 1:** sgRNA sequences used for CLIC4 deletion by CRISPR/Cas9.

	Target exon	Predicted potency	sgRNA
sgNT			ATCGTTTCCGCTTAACGGCG
sg*Clic4* #1	5	0.77	caccgAATCGACGAGAACAGCATGG
			aaacCCATGCTGTTCTCGTCGATTc
sg*Clic4* #2	3	0.58	caccgCTTCAACAGCGAAGTCAAGA
			aaacTCTTGACTTCGCTGTTGAAGc
sg*Clic4* #3	2	0.44	caccgGCCTTACCTTTTCAGGTCAA
			aaacTTGACCTGAAAAGGTAAGGCc
sg*Clic4* #4	3	0.41	caccgGGTCAGATACTCACTTGGGT
			aaacACCCAAGTGAGTATCTGACCc
sg*Clic4* #5	5	0.68	caccgTGTTCTCGTCGATTTCGTCG
			aaacCGACGAAATCGACGAGAACAc
sg*Clic4* #6	1	0.39	caccgTTGACGAAGAGCTCGATGAG
			aaacCTCATCGAGCTCTTCGTCAAc

sgRNA, single guide RNA; NT, non-targeting.

Cell lines were derived from single cell clones and tested *in vivo* to assess primary tumor and metastatic potential. Clones with sg*Clic4* #2 and #6 were selected for further experiments based on their similarity in growth and metastatic potential relative to the parental cells.

### Host genotype influences the primary tumor and lung proteome

**Fig 6:**
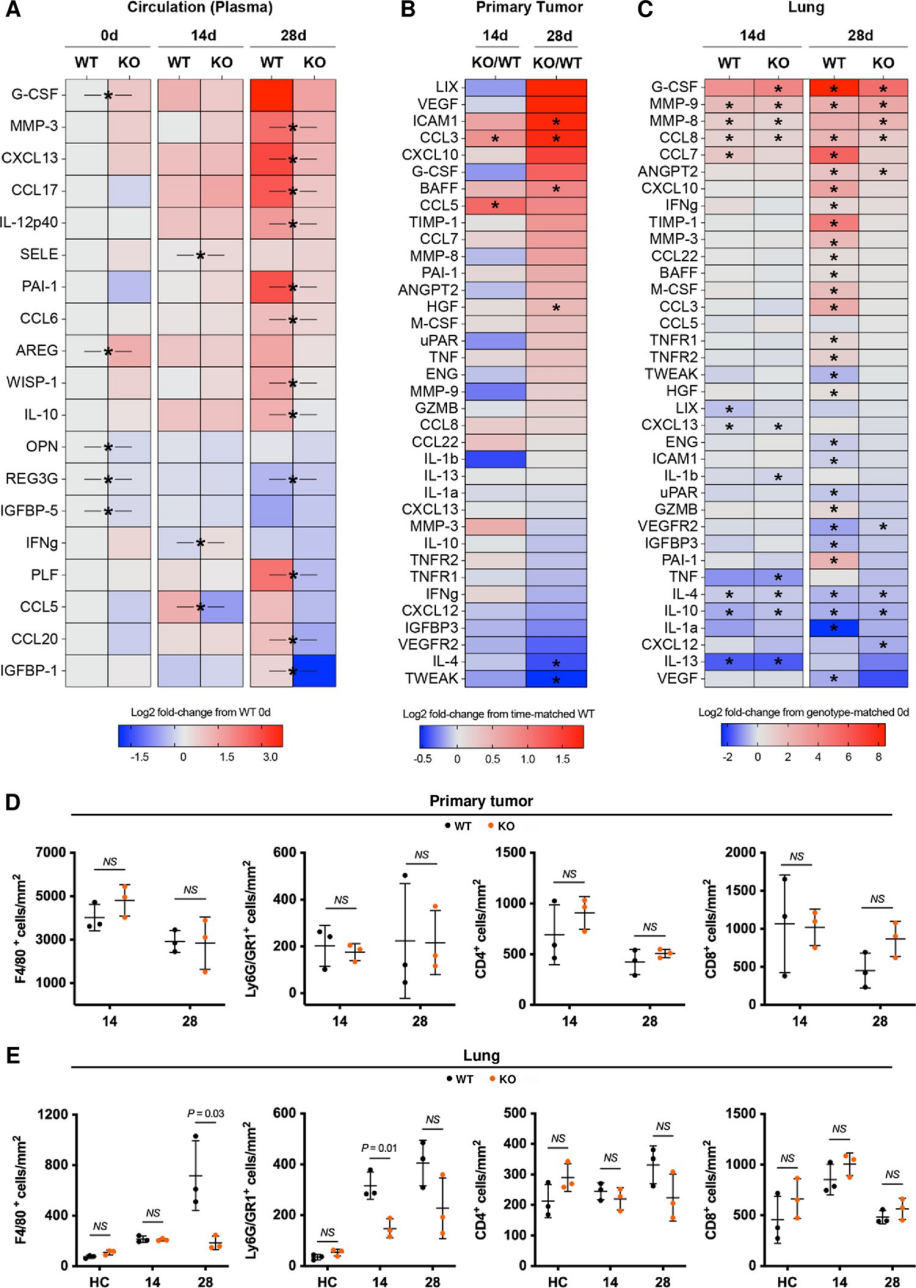
*Clic4* host deficiency alters the circulating and resident proteome of primary tumors and lungs. (*A*) Proteome Profiler Array heat map of 19 significantly differentially expressed cytokines and chemokines, from 111 tested, present in plasma from healthy control (0 days) (n = 3) or 6DT1 tumor-bearing *Clic4* wildtype (WT) or knockout (KO) mice at 14 days (n = 3) or 28 days (n = 5) after mammary fat pad implantation. Raw values were obtained by determining the pixel intensity for each analyte followed by normalization to the corresponding internal control for each animal. These values were log2-transformed, and the heat map represents the mean log2 fold-change of each group from the WT healthy control group. P values were determined by unpaired t test comparing the raw values of WT and KO at each time point; *P value < 0.05. (*B-C*) Luminex Array heat map of 35 significantly differentially expressed proteins, from 51 tested, present in the primary tumors and lungs of *Clic4* wildtype (WT) or knockout (KO) mice at 0 days (lung only, n = 5), 14 days (n = 8), or 28 days (n = 5) after 6DT1 tumor cell implantation in the mammary fat pad. (*B*) Tumor protein expression values shown are log2 fold-change of KO from WT at 14 or 28 days. P values were determined by unpaired t test comparing the raw values of WT and KO at each time point; *P value < 0.05. (*C*) Lung protein expression values shown are log2 fold-change of WT or KO at 14 or 28 days post-implantation from their respective genotype-matched baseline (0 days) values. P values were determined by unpaired t test comparing the raw values of WT or KO at 14 or 28 days to their respective baseline values; *P value < 0.05. (*D-E*) Quantification of selectively stained infiltrating inflammatory cells in primary tumor and lung tissues from 6DT1 tumor-bearing *Clic4* wildtype (WT) and knockout (KO) mice by IHC. Values represent the mean ± s.d. of the total number of positive cells found in tissue sections from 3 animals analyzed by Image Scope. F4/80 was used to detect macrophages, Ly6G/GR1 for neutrophils, and CD4 and CD8 for mature T cell subsets in primary tumors (*D*) and lung tissues (*E*). P values were obtained after unpaired t test between genotypes at each time point.

We used proteomic analyses to identify genotype-relevant distinguishing factors that could influence metastatic competence. A proteomic profiler cytokine array evaluated 111 soluble circulating factors in plasma of 6DT1 tumor-bearing mice of which 15 were statistically different by genotype after 14 and 28 days (Fig 6A). Using the plasma of healthy phenotypically normal control animals as the standard (first column, log2 fold-change = 0), changes in circulating levels of specific factors were detected in untreated mice deficient in CLIC4 (second column), indicating previously undetected constitutive metabolic alterations may be associated with systemic CLIC4 deletion. Further genotype-specific changes were detected in the plasma after introducing a primary tumor in the mammary fat pad for 14 days (e.g., CCL5, IFN-gamma, E-selectin). A reduction in CCL5 at all *Clic4* KO timepoints is particularly notable since CCL5 is reported to influence the development of breast cancer metastasis in human patients and experimental models [39,40]. With enlarging primary tumors in both genotypes and abundant metastases specifically in wildtype but not *Clic4* KO mice at 28 days, major increases in multiple circulating factors (e.g., MMP-3, CXCL13, CCL17, PAI-1, CCL6, CCL20) were measured in WT plasma, constituting potential biomarkers for metastatic colonization. Tissue lysates from primary tumors and lungs of tumor-bearing mice were analyzed by multiplex ELISA (Luminex) (Fig 6B and 6C). Of 51 cytokines, chemokines, and growth factors evaluated on the Luminex array, there were 35 statistically different values by genotype among the primary tumor and lung tissues measured at baseline, 14, and 28 days after tumor implant. Despite implantation of identical 6DT1 cells, CLIC4 deficiency in the host microenvironment led to relative increases in many factors within the primary tumor proteome, including pro-inflammatory cytokines such as CCL3, also known as macrophage inflammatory protein 1-alpha, at both 14 and 28 days (Fig 6B). Additionally, anti-inflammatory IL-4 was significantly lower in the tumors of *Clic4* KO hosts at 28 days (Fig 6B), suggesting net increases in the inflammatory milieu upon CLIC4 loss in the tumor microenvironment. The Luminex analysis also detected genotype-specific responses in lungs to the presence of a distant primary tumor (Fig 6C). At 14 days, which is prior to metastatic residence but during metastatic niche development, the lungs of both genotypes recognized the presence of a primary tumor by modulating the expression of proteases (MMP8 and 9) and cytokines (CCL8, CXCL13, IL-4, IL10, IL-13) relative to their genotype-matched control lungs. Genotype-specific differences (G-CSF, IL-1b, TNF) were also detected at this timepoint. Consistent with the pattern of circulating factors, by 28 days, when the WT lungs were laden with metastases, the burden of cytokines, chemokines, and growth factors was much greater in WT lungs relative to their normal lung controls, while the relative profile from CLIC4-deficient metastasis-free lungs had changed little from the day 14 pattern in tumor-bearing mice, with the exception of ANGPT2 and VEGFR2. *In situ* analysis of primary tumors and lungs with immunostaining showed little variation among infiltrating neutrophil (Ly6G/GR1-positive cells) or macrophage (F4/80-positive cells) populations (Fig 6D) by genotype, although the level of macrophage infiltrate was high in tumors from both genotype hosts. Likewise, T cell content (CD4- or CD8-positive cells) in the primary tumor (Fig 6D) did not significantly differ at any timepoint by host genotype even as tumors continued to expand during this period. The introduction of a primary tumor elevated neutrophil counts in the lungs of both genotypes at 14 and 28 days but that response was less robust in the *Clic4* KO hosts (Fig 6E). Only the lungs of WT mice at 28 days, which were filled with metastatic nodules, were highly infiltrated by macrophages (Fig 6E). The lungs of neither genotype evoked a significant T cell response at any timepoint even when the WT lungs were burdened with metastases (Fig 6E).

### Host genotype influences primary tumor and lung transcriptome

**Fig 7:**
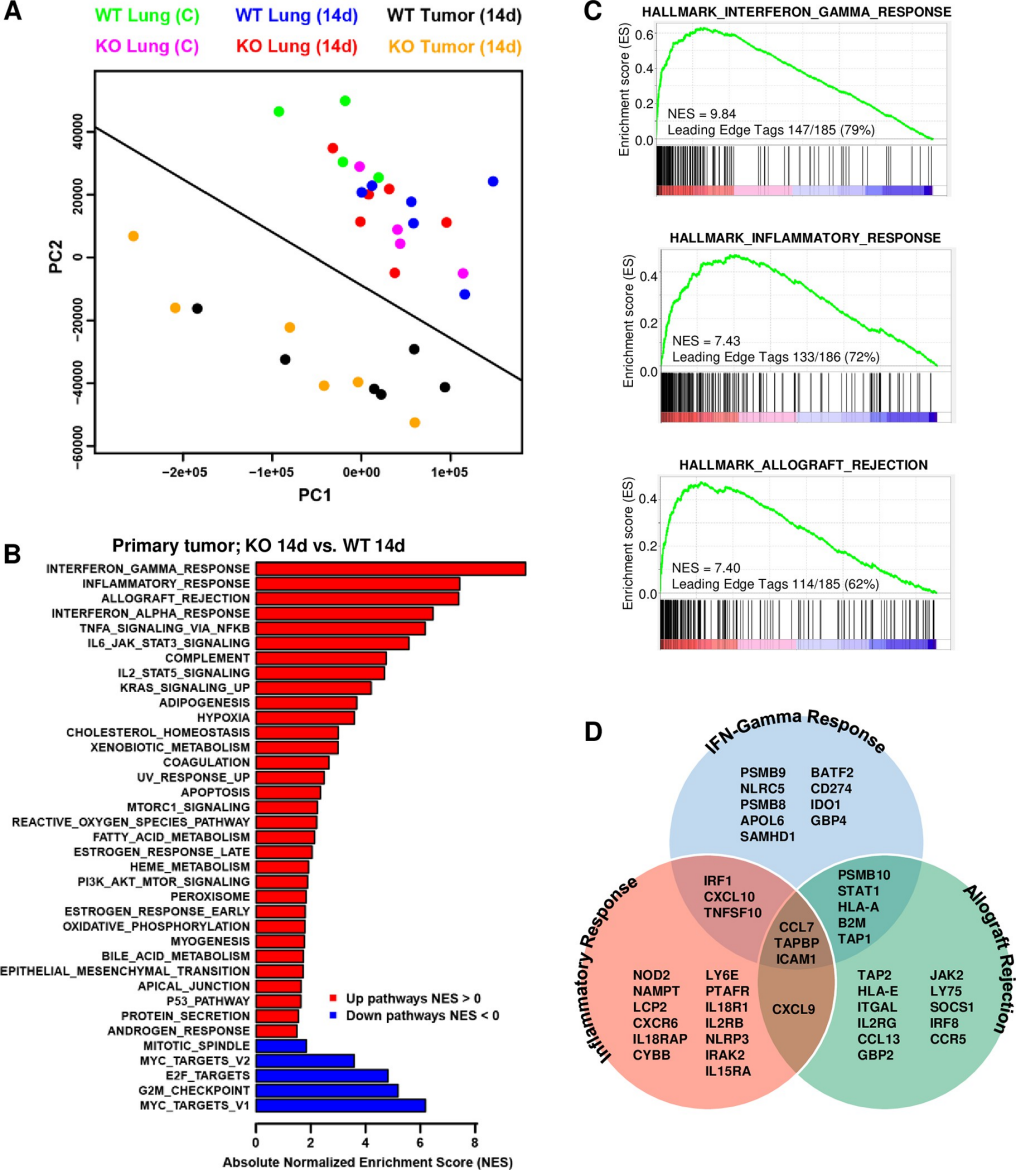
RNA-seq analysis reveals that host genotype determines the transcriptome of primary tumors implanted for 14 days. (*A*) Principal component analysis (PCA) of n = 32 RNA-seq samples from normal WT and KO lungs (n = 8), lungs from WT and KO tumor-bearing mice (n = 12), and primary tumors from WT and KO mice (n = 12) at 14 days post-implantation shows separation of samples based on tissue of origin but not genotype. (*B*) Gene set enrichment analysis (GSEA) interrogated MSigDB Hallmark gene sets to determine if genes differentially expressed between KO and WT tumors were significantly enriched in distinct biological pathways. Only significantly (FDR <0.1) upregulated (red) and downregulated (blue) pathways are shown, represented by absolute normalized enrichment score (NES). (*C*) Representative enrichment plots of the top three upregulated pathways show the frequency of leading-edge genes contributing to the NES. (*D*) Venn diagram showing the overlap between the first 20 leading-edge genes from the top pathways upregulated in KO tumors, Interferon-Gamma Response, Inflammatory Response, and Allograft Rejection.

We next performed RNA sequencing of tissues at premetastatic stages to identify possible transcriptional changes that might influence the metastatic process. RNA from control lungs and 14-day lungs and primary tumors of 4–6 independent animals of each genotype was interrogated. The premetastatic 14 day timepoint was considered the optimal time to detect host-mediated environmental changes permissive or restrictive for metastatic progression. Based on FPKM expression of all genes, principal component analysis (PCA) showed linear separation of primary tumor from lung but no separation among the lung samples by genotype or tumor burden (Fig 7A). A heat map was assembled from the 100 most differentially expressed genes at 0.05 FDR and 2-fold change from the DESeq2 analysis comparing the primary tumors growing for 14 days from *Clic4* KO mice and those from WT mice (S3 Fig). Based on the DESeq2 analysis of the primary tumor data, we applied GSEAPreranked to interrogate Hallmark pathways, which yielded normalized enrichment scores (NES) plotted as *Clic4* KO mice relative to WT mice (Fig 7B). Top NES scores illuminated inflammatory and allograft rejection pathways with leading edge genes (Fig 7C and 7D) encoding overlapping proinflammatory cytokines and related gene families. Notably, *CCL7*, *TAPBP*, and *ICAM1* were leading edge genes for all three pathways, and both CCL7 and ICAM1 were also trending up in the 14-day primary tumor proteomic analysis (Fig 6B). To infer immune cell composition in primary tumors, we applied CIBERSORT [41] using the ImmuCC signature gene matrix of 511 genes [42]. PCA analysis using the genes from this signature showed distinct separation of tumors from each host genotype (S4A Fig). 40 of the genes differentially expressed between KO and WT tumors intersected with the ImmuCC gene signature (p = 3.5e-30) (S4B Fig) and are shown as a heat map in S4C Fig. These signatures align with relative increases in IL-17-secreting T cells and M1 macrophages (S4D Fig) in primary tumors from the *Clic4* KO host, suggesting these tumors reside in a genotype specific inflammatory microenvironment.

**Fig 8:**
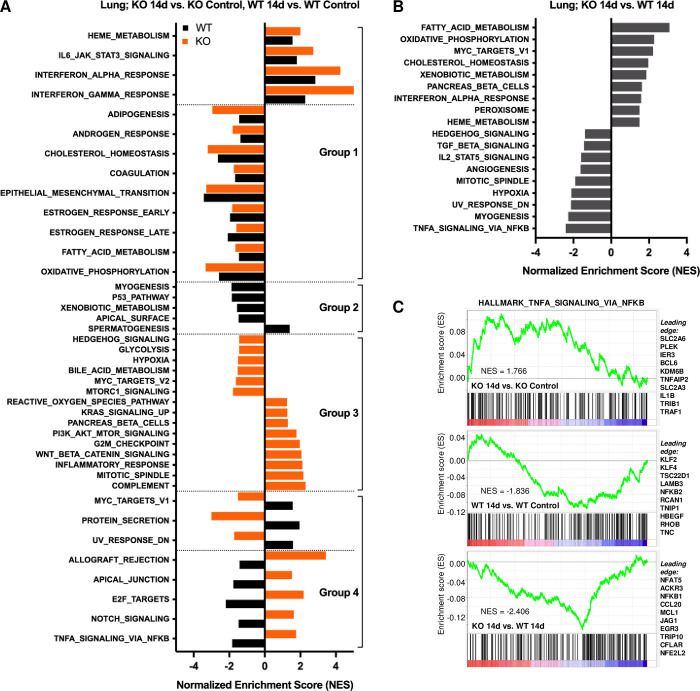
RNA-seq reveals that host genotype determines the consequences of a distant primary tumor on the lung transcriptome. (*A*) Significant (FDR <0.25) Hallmark pathways identify transcriptomic changes that occur in the lungs of 6DT1 tumor-bearing *Clic4* wildtype (WT) or knockout (KO) mice relative to the lungs of genotype-matched healthy control mice 14 days after implantation. Gene set enrichment analysis (GSEA) normalized enrichment scores (NES) are shown. Group 1, similar regulation between WT and KO; Group 2, significant for WT only; Group 3, significant for KO only; Group 4, opposite regulation between WT and KO. (*B*) Gene set enrichment analysis (GSEA) to identify significant enrichment (FDR <0.25) in Hallmark pathways based on genes differentially expressed in KO vs. WT lungs at 14 days after primary tumor implantation. (*C*) Enrichment plots for the “TNFA signaling via NFKB” Hallmark pathway for the three comparisons shown in panels A and B. The ten most significant leading edge genes are listed to the right of each plot.

Gene expression differences were also evaluated in the 14-day premetastatic lungs to identify specific transcriptional changes that distinguish naïve lungs from lungs of tumor-bearing mice prior to metastasis for each genotype. This approach defines genotypic differences in the transcriptome of the lung premetastatic niche in response to a distant primary tumor. We first confirmed that expression of other CLIC4 paralogs did not compensate as a consequence of CLIC4 loss (S5A Fig). *Clic4* FPKM reads detected in KO lungs represent mapped RNA reads outside of the region surrounding exon 2 excised in the KO mouse [18], but CLIC4 protein was not detected in isolated KO lung fibroblasts (S5B Fig). Heat maps of differentially expressed genes were developed from premetastatic lungs of tumor-bearing and naïve mice from the same genotype, with a greater number of significant changes detected in the lungs of WT than KO mice at 14 days after primary tumor implantation (S5C and S5D Fig). The nature of these changes was interrogated using GSEA analysis to determine enrichment in Hallmark pathways (Fig 8A). While several pathways were similarly upregulated or downregulated in the lungs of WT and KO mice (interferon, epithelial mesenchymal transition, oxidative phosphorylation) (Fig 8A, Group 1) and may indicate a general transcriptional response of lungs detecting the presence of a primary tumor, other pathways were uniquely altered or regulated in the opposite direction between genotypes. Particularly noteworthy as metastasis relevant was the increase in inflammation-related pathways specifically in the KO lungs (inflammatory response, complement, reactive oxygen species) (Fig 8A, Group 2). In contrast, gene sets unique to WT lungs identified pathways reported to be downregulated in circumstances of tissue damage (apical surface, xenobiotic metabolism, p53 pathway) (Fig 8A, Group 3). Other pathways (allograft rejection, TNFA signaling via NFKB) were upregulated in KO but downregulated in WT (Fig 8A, Group 4). We also directly compared the premetastatic lungs of tumor bearing mice of each genotype and identified relative reductions in two established CLIC4-regulated pathways, angiogenesis and TGF-β signaling, among the pathways that distinguished *Clic4* KO from WT lungs (Fig 8B). This direct comparison of primary tumor-activated lungs across genotypes also confirmed a KO lung environment increased in interferon and oxidative phosphorylation pathway genes. Interestingly, we observed negative enrichment for the TNFA signaling via NFKB pathway when comparing KO to WT premetastatic lungs (Fig 8B), but positive enrichment when comparing premetastatic KO to naïve KO lungs (Fig 8A, Group 4). Further investigation of the genes contributing to the enrichment scores for each comparison showed limited overlap, with the ten most significant leading edge genes for each comparison being completely unique (Fig 8C). These differences emphasize the importance of the context of comparative transcriptomics and conclude that host CLIC4 status is a determinant of the microenvironment of a primary tumor and the response of the lungs to the presence of the primary tumor.

## Discussion

From discoveries made in mouse models, we have linked *CLIC4* expression to clinical parameters that define outcome in human breast cancer. *CLIC4* expression is higher in breast cancers from young patients whose tumors are often more aggressive [43], in patients with triple negative breast cancer, those with local metastases and with poor prognosis by Kaplan-Meier analysis. Emerging evidence suggests that elevated *CLIC4* expression is a marker for poor outcome at other cancer sites [5]. Previous *in situ* studies have shown that elevation of CLIC4 in human cancers is particularly prominent in host stromal cells [9], consistent with our analysis of progressing breast cancer (Fig 1A) and cancers of the cervix, esophagus, and tongue [27]. Experimentally, elevation of stromal CLIC4 is tightly linked to TGF-β signaling [12,13,22], a pathway causally associated with breast cancer progression and metastasis [44]. We now provide human proteomic data indicating that CLIC4 expression levels and those of members of the TGF-β pathway in breast cancer patients are highly correlated implying that CLIC4 levels could serve as a surrogate TGF-β effector of TGF-β-driven pathologies.

The near total inhibition of metastasis by *Clic4* depletion in the host suggests that fundamental host requirements for metastatic competence, irrespective of tumor heterogeneity and high EMT, have been modified. Other studies initiated in mice revealing host genes that impact metastatic competence in breast cancer include *Klf4* [45], *Arntl2* [34], and *Zbtb16* [46], [47] also have clinical associations. However, in the experimental murine setting, CLIC4 appears unique in the near total dependence of metastasis on its protein functions. Since host cells are the target in this study, in contrast to the usual focus on tumor-targeted therapy, a therapy developed to inhibit CLIC4 function in the host would be less likely to develop resistance as occurs in targeting heterogeneous and genomically unstable cancer cells. Currently, anti-TGF-β antibody therapy is under study as a clinical approach to inhibit breast cancer progression [48]. If CLIC4 is an effector of TGF-β pro-metastatic activity, it represents a more selective and proximal target for therapeutic intervention.

We now are challenged to illuminate the CLIC4 functions that enhance metastatic competence in the host. An extensive search of available databases failed to identify recurrent *CLIC4* structural genomic alterations associated with any human malignancy including breast cancer [27]. Experimental data indicate that stromal cells expressing high levels of CLIC4 stimulate the growth of xenografts or cultures of human breast cancer cells [12,13,21,22,49]. Genomic and proteomic analyses and experimental studies in our murine models have identified inflammatory pathways associated with host CLIC4 depletion that could impact metastatic spread. Previous disparate observations of CLIC4 actions in isolated systems have also contributed some insights on important CLIC4 functions that when consolidated, could have substantial impact on a biological process as complex as metastasis. In our study CLIC4 contributed to the normal development of blood vessels in the primary tumor. In isolated systems CLIC4 was required for neo-angiogenesis [14], vascular morphogenesis [17], formation of collateral vessels [15], and endothelial tube formation [14,16]. These functions may have contributed to necrosis in the primary tumor and reduced expression of genes involved in angiogenesis in the premetastatic lungs of CLIC4-deficient mice (Fig 8B). Other functions of CLIC4 relevant to metastasis have been reported in specific models and include adhesion of cells to extracellular matrix, integrin activation, and cytokine release [20,50,51]. We found evidence that CLIC4-deficient tumor-bearing mice differed from WT mice in the pattern of circulating factors, some of which are associated with migration and adhesion of leukocytes. Previous reports showed that CLIC4-deficient mice had lower circulating levels of CCL5 than controls after LPS treatment [50], a finding we now extend to plasma of tumor-bearing mice. Multiple studies indicated that circulating CCL5 promotes breast cancer metastasis [39,40,52–54], so this connection warrants further study. Thus, the effectiveness of a multifunctional protein like CLIC4 to inhibit a multifactorial process such as metastasis may just lie in its precise array of activities in required pathways.

Our proteomic and transcriptomic analyses yielded an unrecognized consequence of host CLIC4 loss for enhancing the inflammatory milieu of the primary tumor site. These studies also showed that the distant target organ for metastatic spread responded to a primary tumor elsewhere with a pattern of inflammation-related changes that differed by genotype. *In situ* studies did not reveal substantial differences in numbers of infiltrating CD4^+^ T cells, CD8^+^ T cells and Ly6G-GR1^+^ neutrophils in the primary tumor or the premetastatic lung of CLIC4 KO and WT mice. The Human Protein Atlas shows that CLIC4 is expressed to varying levels in immune cells [55], and while our *in situ* analysis yielded important localization information, it did not capture CLIC4-regulated activation or polarization states of the infiltrating cells. It did confirm that a massive influx of inflammatory cells was not the basis for lack of metastasis in CLIC4-deficient hosts. More likely, CLIC4 is needed to modify the functions of infiltrating and resident host cells to create a more receptive environment. For example, our CIBERSORT analysis identified enrichment for macrophage M1 polarization in primary tumors in the KO host, suggesting qualitative but not quantitative differences of tumor-infiltrating macrophages in CLIC4-deficient animals. We have shown that CLIC4 is necessary for macrophages to progress out of the M1 phenotype upon activation [56] and CLIC4 is required for the formation of neutrophil extracellular traps essential for angiogenesis [57]. The loss of these CLIC4 functions could contribute to the altered immune milieu of primary tumor and lung in *Clic4* KO hosts detected by transcriptomic analysis. Reports of CLIC4 involvement in the pathogenesis of several human autoimmune diseases, including allergic asthma and scleroderma [7,8], add to an expanding awareness of CLIC4 as a mediator of immune function. The factors that contribute to metastatic incompetence as a consequence of loss of CLIC4 in the host are summarized graphically in S6 Fig.

The question remains as to what molecular function or functions of CLIC4 regulate the phenotype currently illuminated. The oxidoreductase activity of CLIC1, 3, and 4, first shown by Al Khamici et al. [19], could contribute to redox regulation in normal and cancer cells and have broad effects. In fact, elevated reactive oxygen species was identified in the GSEA analysis of both the primary tumor and the premetastatic lungs of CLIC4 KO mice. Redox signaling can impact multiple cellular processes, including cancer progression. For example, the release of CLIC3 from both tumor and stromal cells into the tumor microenvironment stimulates the invasion of endothelial cells and invasive pseudopods from breast cancer cells through its oxidoreductase activity on transglutaminase-2 that modifies the stiffness of the extracellular matrix [58]. While secretion of CLIC4 *in vivo* has not been documented, its release in extracellular vesicles could influence local and distant sites of tumor growth. CLIC proteins also have putative ion transport functions in artificial and biological membranes, although the specificity of their action is still debated [4]. In a recent report, CLIC1 was shown to interact with the potassium channel EAG2 to promote the growth of medulloblastoma by regulating cell volume, identifying CLIC1 and EAG2 as potential therapeutic targets [59]. Illuminating all the underlying mechanisms associated with the substantial loss of metastatic competence associated with the deficiency of host CLIC4 will require additional investigation.

The modes of CLIC4 action, both now identified and others yet to be elucidated, will likely require mutagenesis studies, biophysical analyses, and additional model systems to fully understand its contribution to the human diseases to which it is increasingly attached. Elevated CLIC4 levels in plasma of several human diseases, including progression of ovarian cancer [10] and the pathogenesis of pulmonary hypertension [6], suggest the protein may have systemic as well as local cellular effects. The concept of treating the host microenvironment to prevent cancer spread is gaining attention [60] as an extension of the concept of immunotherapy. Targeting the host factors contributing to the development or maintenance of cancer metastases will require new classes of drugs or genetic approaches. Using preclinical models with clinical correlates, we have identified CLIC4 as a potential host factor to be exploited for therapy, and we have exposed pathways through which CLIC4 contributes to metastatic competence. We anticipate that this work will stimulate drug development to push this concept further.

## Materials and methods

### Ethics statement

The research described in this study was performed under Animal Study Protocol LCBG-035, approved by the National Cancer Institute (NCI) Animal Care and Use Committee, in accordance with all institutional ethical guidelines.

### Cells and cell culture

The murine breast cancer cell line 6DT1 (FVB background) was grown as previously described [61] and E0771 (C57BL/6 background) cells were purchased from CH3 BioSystems LLC and grown according to the manufacturer’s recommendations (Amherst, NY, No. 940001). Primary fibroblasts from dissociated murine lungs were isolated and cultured in complete Fibroblast Medium (ScienCell, Carlsbad, CA, No. 2301).

### CLIC4 deletion by CRISPR/Cas9

Parental 6DT1 cells were infected with lentiviral particles containing CRISPR/Cas9 and sgRNA targeting murine *Clic4* (Table 1) at a multiplicity of infection (MOI) of 1 in medium containing polybrene (4 μg/ml; Sigma-Aldrich). Cells containing the sg*Clic4* were selected by puromycin. *Clic4* deletion was confirmed by western blot in a total of six different sgRNAs.

### Histology and immunohistochemistry

Human breast cancer tissue microarrays were purchased from US Biomax, Inc (Rockville, MD, BR1503g, BR2082 b, BR20837a, BRC481). For analysis and quantification of metastatic lesions, lungs were insufflated with 10% NBF (neutral buffered formalin, ThermoFisher, Waltham, MA) prior to their removal. Tissues were fixed in 10% NBF overnight and transferred to 70% ethanol until processing. Paraffin sections were made by Histoserv Inc. (Germantown, MD). Tissue sections were deparaffinized by immersion in HistoChoice (Amresco, Inc., Solon, OH) for 10 minutes, rehydrated followed by antigen retrieval with citrate buffer pH 6.1 (Target Retrieval Solution 10X, Dako, Agilent, Santa Clara, CA). Tissue sections were washed and quenching of endogenous peroxidase was performed by 3% hydrogen peroxide for 5 minutes. Slides were blocked for 30 minutes with serum-free protein block (Dako, Agilent, Santa Clara, CA). The following antibodies were purchased from Cell Signaling Technology Inc, Beverly, MA: CLIC4 No. 12644, 1:200; GFP No. 2555, 1:200; F4/80 D2S9R No. 70076, 1:200; CD31 No. 77699, 1:100; Vimentin, No. 5741, 1:200. Additional antibodies used were CD4, No. 13–9766, 1:250 and CD8a No. 14-0195-82, 1:50 both from eBioscience/ThermoFisher, Waltham, MA, and Ly6G/GR1 DM3589, 1:100 from Acris Origene, Rockville, MD. Antibodies were diluted in 3% BSA solution and incubated overnight at 4°C. After washing, slides were treated for 1 hour with the corresponding biotinylated secondary antibody (ThermoFisher, Waltham, MA, 1:200) diluted in 3% BSA solution followed by avidin-biotinylated conjugation using the ABC system (Vector Labs, Burlingame, CA) for 30 minutes. ImmPACT DAB (Vector Lab, Burlingame, CA) was used for staining development following the manufacturer suggestions. Counterstaining was performed with Hematoxylin QS (Vector Lab, Burlingame, CA) for 1 minute and water wash. All slides were dehydrated and mounted with VectaMount (Vector Lab, Burlingame, CA). Tissue sections were also stained by hematoxylin and eosin to quantify metastatic load and scanned and analyzed using a ScanScope XT and Aperio ImageScope viewing software version 12.3.3 (Leica Biosystems Imaging Inc, Buffalo Grove, IL). The Pathology/Histotechnology Laboratory of the National Cancer Institute, Frederick, MD, performed IHC staining for F4/80, CD4, CD8a, and Ly6G/GR1.

### Digital image analysis of vimentin staining

Scanned digital image data files were created as whole slide images of 3 independent primary tumors of each genotype after 14 days using the Leica AT-2 digital slide scanner at a resolution of 0.50 μm/pixel (20× objective). Scanned slide images were imported into HALO imaging software. Slides were manually annotated using the HALO pen drawing tool to segment the tumor tissue area to be analyzed based upon visual assessment of the mass lesion. A unique machine learning pattern classifier algorithm was created using study specimens to designate areas within the tumor tissue segmented image area to classify tumor, tumor necrosis, loose areolar connective tissue (fat), or glass. Data output included area measurements for each tissue class for respective specimens. HALO Area Quantification algorithm was programed with appropriate intensity thresholding for the existing vimentin intermediate filament IHC chromogenic signal development, and subsequently used to quantify the vimentin expression in the tumor as seen by the brown DAB signal in each tissue section. Image analysis QA was performed by visual inspection on post processed image markup files.

### Immunoblotting

Protein lysates were prepared from cultured cells in RIPA buffer (Cell Signaling Technology Inc, Beverly, MA, No. 9806), supplemented with EDTA-free Halt protease and phosphatase inhibitors (Thermo Scientific, Waltham, MA, No. 1861281). Protein concentration was measured using BCA or microBCA kits (Thermo Scientific, Waltham, MA, No. 23225 and No.23235). Between 3 to 10 μg of protein lysate was loaded in precast 4–20% Tris-Glycine protein gels (Bio-Rad Laboratories No. 5671094 and No. 5671095), transferred to a nitrocellulose membrane (Bio-Rad, No. 1620168), and probed with the appropriate primary and secondary HRP-conjugated antibodies. Blots were developed by chemiluminescence substrates (Thermo Scientific, No. 34078 and No. 34076) using a ChemiDoc Imager (Bio-Rad). Immunoblots were performed with antibodies against CLIC4 (Cell Signaling, No. 12644), CLIC1 (Cell Signaling, No. 53424), and HSP90 (Cell Signaling, No.4877).

### *In vivo* breast cancer models

The construction of the *Clic4* KO mice on a mixed background was reported previously [18]. *Clic4* KO mice were backcrossed onto pure FVB/N or C57BL/6 backgrounds and purity was confirmed by microsatellite sequencing. For experiments, heterozygous *Clic4* KO mice were intercrossed and virgin female wildtype and/or *Clic4* KO littermates were used at six- to eight-weeks of age. 1 × 10^5^ 6DT1 or 2.5 × 10^5^ E0771 cells were injected orthotopically into the fourth mammary fat pad of syngeneic FVB/N or C57BL/6J mice, respectively. Animals were euthanized between 7 to 28 days after implantation, and primary tumor, lungs, and whole blood in heparinized tubes were collected. Primary tumors were weighed, and the number of superficial lung metastases were counted. Tissues were either fixed in 10% NBF or snap-frozen in liquid nitrogen for further evaluation. Plasma was obtained after centrifugation of heparinized whole blood for 20 minutes at 3,000 rpm at 4°C and kept at -80°C until use.

For tail vein injection, 14 days post-implantation of primary tumor, wildtype or *Clic4* KO FVB/N females were injected with 1 × 10^5^ 6DT1 cells expressing GFP into the lateral tail vein. Mice were euthanized 14 days post-tail vein injection. All procedures were performed under the Animal Safety Protocol (LCBG-035) and approved by the NCI-Bethesda Animal Care and Use Committee.

### Proteome cytokine array

Plasma from healthy controls or tumor-bearing mice were used to evaluate circulating mediators by using the Proteome Profiler Mouse XL Cytokine Array (R&D Systems, Inc, Minneapolis, MN, No. ARY028) following the manufacturer’s instructions. Relative pixel intensity of duplicated spots from each membrane were analyzed using HLImage++/Quick Spots image analysis software (Western Vision Software, Salt Lake City, UT).

### Luminex chemokine array

Primary tumor or lung tissues were harvested and kept at -80°C until lysis in 1X RIPA buffer supplemented with phosphatase/protease inhibitors (Cell Signaling) using a Precellys 24 tissue homogenizer (Bertin Instruments, MD, USA). Protein concentration was measured using BCA or microBCA kits (Thermo Scientific, Waltham, MA, No. 23225) and 50 μg of total protein were used to analyze 51 factors using Mouse Magnetic Luminex Assays in a format of 24- and 27-plex panels (R&D Systems, Inc, Minneapolis, MN) following the manufacturer’s recommendations.

### RNA isolation and sequencing

Pre-metastatic lungs of female FVB/N mice implanted with 6DT1 cells in the mammary fat pad for 14 days were harvested, separated into five lobes, snap-frozen in liquid nitrogen and stored at -80°C until use. The primary tumors were harvested and frozen at the same timepoint. Total RNA was isolated using the RNeasy kit (Qiagen). mRNA sequencing was performed using Illumina Sequencing Technology by the Sequencing Facility at the Frederick National Laboratory for Cancer Research (FNLCR). Raw fastq files were aligned and mapped to the GRCm38(mm10) mouse reference genome by STAR (Version 2.7.8a) and the expression levels of genes and transcripts were quantified by using RSEM (Version 1.3.2). The DESeq2 R package was applied for differential expression analysis comparing samples in different conditions including comparisons of 14-day primary tumors KO vs. WT, 14-day lung samples KO vs. WT, and 14-day lung samples KO vs. normal control. Significant Hallmark pathways were found by using GSEAPreranked after ranking the genes by -log10(p-value)*(log2 fold-change sign) based on the p-value and log2 fold-change statistics from the DESeq2 analysis. To show the expression of some sets of differentially expressed genes by heatmaps, we first transformed the FPKM values by the function log2(1+x), and then computed the cross-sample z-statistics. The PCA projections of the samples included those using all genes and those using the CIBERSORT signature genes. The data discussed in this publication have been deposited in NCBI’s Gene Expression Omnibus [62] and are accessible through GEO Series accession number GSE185163 (https://www.ncbi.nlm.nih.gov/geo/query/acc.cgi?acc=GSE185163).

### Human data validation

The METABRIC and TCGA-BRCA datasets were used to examine the prognostic value of the *CLIC4* expression for breast cancer patients. The RNA expression data for n = 1082 samples in the TCGA breast cancer dataset was downloaded from cBioPortal. Computation of Spearman correlations between *CLIC4* and nine TGF-β genes were computed: *TGFB1*, *TGFB1I1*, *TGFB2*, *TGFB3*, *TGFBI*, *TGFBR1*, *TGFBR2*, *TGFBR3*, *and TGFBRAP1*; six of them had a strong positive Spearman correlation with the Spearman rho values greater than 0.2 and p-values less than 3e-11. For Proteomic datasets, two studies were available from the NCI Proteomic Data Commons: TCGA Breast Cancer Proteome with n = 108 cases and Prospective Breast BI Proteome with n = 125 cases were evaluated for the same correlations found in gene expression. The protein TGFBR2 had 81% missing values in the first dataset and had no values in the second one. We combined the two protein datasets and calculated the Spearman correlations after excluding TGFBR2. Six out of the eight TGF-β family members had the Spearman rho values greater than 0.17 and p-values less than 8e-3. The four most significant genes, TGFB1, TGFB1I1, TGFB2, and TGFBI had the Spearman rho values greater than 0.37 and p-values less than 5e-10. Three genes, TGFB1I1, TGFB2, and TGFBI were also significantly correlated with CLIC4 based on both RNA and protein datasets.

### Statistics

Graphs and statistical analyses were performed using R and Prism software (GraphPad Software Inc.). Data were expressed as mean ± s.d. For analysis of three or more groups, analysis of variance (ANOVA) tests were performed with a Bonferroni or Tukey post-hoc test, when appropriate. Analysis of differences between two normally distributed test groups was performed using the Student’s t-test. P values were considered statistically significant if *P* < 0.05.

## Supporting information

S1 Fig Several datasets show that expression of CLIC4 and TGF-β pathway proteins are highly correlated. **A** METABRIC and TCGA-BRCA breast cancer datasets, with 75th percentile as the cut-off and 25-year censored data, show the high *CLIC4* group had poor survival compared to the low *CLIC4* expression group compared by the Kaplan-Meier curves in the first 10 years. **B-E** Correlation between CLIC4 and the four most significant proteins, TGFB1 (**B**), TGFB1I1 (**C**), TGFB2 (**D**), and TGFBI (**E**) with the Spearman rho values greater than 0.37 and p-values less than 5e-10. Datasets from the TCGA Breast Cancer Proteome with n = 108 cases and the Prospective Breast BI Proteome with n = 125 cases were combined. The Spearman correlations between CLIC4 and eight TGF-β pathway components were calculated. Six out of the eight proteins were significant with the Spearman rho values greater than 0.17 and p-values less than 8e-3.
(PDF)

S2 Fig Orthotopic grafting of 6DT1 cells in the mammary fat pad yields consistent primary tumor growth and reproducible lung metastases. **A** Immunoblot showing CLIC4 protein expression in the 6DT1 and E0771 cells used in this study. **B** Primary tumor weight (g = grams) and lung metastasis quantification at 14, 21, and 28 days after implantation of 1×10^5^ 6DT1 cells into the fourth left mammary fat pad of FVB female mice. **C-D** Representative hematoxylin and eosin staining (**C**) and CLIC4 immunohistochemistry (**D**) of primary tumor and lung tissues at 14, 21, and 28 days. Whole tissue (left, scale bar = 3 mm) and high magnification (right, scale bar = 10 μm) images are shown.
(PDF)

S3 Fig RNA-seq analysis reveals numerous differentially expressed genes in primary tumors of *Clic4* knockout vs. wildtype host mice. Top 100 most significant (FDR <0.05, fold-change >2) differentially expressed genes in 6DT1 primary tumors from *Clic4* knockout (KO) hosts compared to those from wildtype (WT) hosts at 14 days after mammary gland implantation. For each gene, FPKM values were transformed using the function log2(x+1) and the z-statistic was computed across the 12 tumor samples.
(PDF)

S4 Fig Immune cell composition inferred from RNA-seq analysis suggests that immune cell differentiation or recruitment is altered in tumors of *Clic4*-deficient hosts. The ImmuCC signature genes (42) were compared to the genes differentially expressed (FDR <0.05, fold-change >2) between the tumors derived from *Clic4* wildtype (WT) and those from knockout (KO) host mice. **A** Principal component analysis (PCA) show distinct separation of the tumors from *Clic4* wildtype (WT) and knockout (KO) mice based on the expression of the ImmuCC signature genes. **B** Venn diagram depicting the overlap between the tumor differentially expressed genes (DEGs) and the ImmuCC signature genes. Forty of the 186 DEGs overlapped the ImmuCC signature, a significant overlap with a p-value of 3.5e-30. **C** Heat map of the 40 genes represented by the overlap in (**B**). **D** Three imputed cell fractions, monocyte, M1 macrophage, and Th17, were significantly different in the tumors of *Clic4* wildtype (WT) and knockout (KO) host mice. P values were computed using the Wilcoxon rank sum test.
(PDF)

S5 Fig RNA-seq analysis reveals unique gene expression changes in the lungs of *Clic4* knockout vs. wildtype host mice at pre-metastatic timepoints. **A** mRNA expression of Clic family members by RNA-seq analysis of lungs from control and tumor-bearing *Clic4* wildtype (WT) or knockout (KO) mice 14 days post-implantation of 6DT1 cells, n = 4 lungs per control group; n = 6 lungs per tumor-bearing group. Values are expressed as the FPKM mean ± s.d. **B** Lack of CLIC4 protein expression in *Clic4* knockout (KO) animals was confirmed by Western blot of protein extracted from primary lung fibroblasts of *Clic4* wildtype (WT) or KO mice. **C** Significantly (FDR <0.25, fold-change >1.5) differentially expressed genes in *Clic4* knockout (KO) lung tissue at 14 days-post tumor implantation vs. control lung tissue. **D** Significantly (FDR <0.25, fold-change >1.5) differentially expressed genes in *Clic4* wildtype (WT) lung tissue at 14 days-post tumor implantation vs. control lung tissue.
(PDF)

S6 Fig Deletion of CLIC4 from the mouse genome prevents lung metastasis of implanted breast cancer cells. Broad summary of genotype-dependent differences in the primary tumor, circulation, and lungs of *Clic4* wildtype (WT) and knockout (KO) mice at 14 and 28 days after tumor cell implantation. Based on proteomic and transcriptional data, primary tumors growing in a CLIC4-deficient microenvironment display enhanced necrosis, reduced angiogenesis, higher reactive oxygen species (ROS), and a more active inflammatory milieu (↑ M1 macrophages, INFg, IL6, STAT3, CCL3, CCL5). Differences in plasma circulating factors are also apparent at 14 days. Genotype-dependent differences in the lung milieu at 14 days are similar to those detected in the primary tumor. Lung inflammation and ROS are increased, while angiogenesis and TGF-β pathway signaling are reduced. These differences reflect a common host response to CLIC4 absence in the presence of a primary tumor, but which are responsible for the large difference in metastatic competence has yet to be determined. (Created with BioRender.com).
(PDF)
