# Supplementary material for: Host CLIC4 expression in the tumor microenvironment is essential for breast cancer metastatic competence
Source: PLoS Genet. 2022 Jun 21;18(6):e1010271. doi: 10.1371/journal.pgen.1010271 (PMC9249210; doi:10.1371/journal.pgen.1010271)
Supplement: S1 Fig — A METABRIC and TCGA-BRCA breast cancer datasets, with 75th percentile as the cut-off and 25-year censored data, show the high CLIC4 group had poor survival compared to the low CLIC4 expression group compared by the Kaplan-Meier curves in the first 10 years. B-E Correlation between CLIC4 and the four most significant proteins, TGFB1 (B), TGFB1I1 (C), TGFB2 (D), and TGFBI (E) with the Spearman rho values greater than 0.37 and p-values less than 5e-10. Datasets from the TCGA Breast Cancer Proteome with n = 108 cases and the Prospective Breast BI Proteome with n = 125 cases were combined. The Spearman correlations between CLIC4 and eight TGF-β pathway components were calculated. Six out of the eight proteins were significant with the Spearman rho values greater than 0.17 and p-values less than 8e-3. (PDF) [file pgen.1010271.s001.pdf]

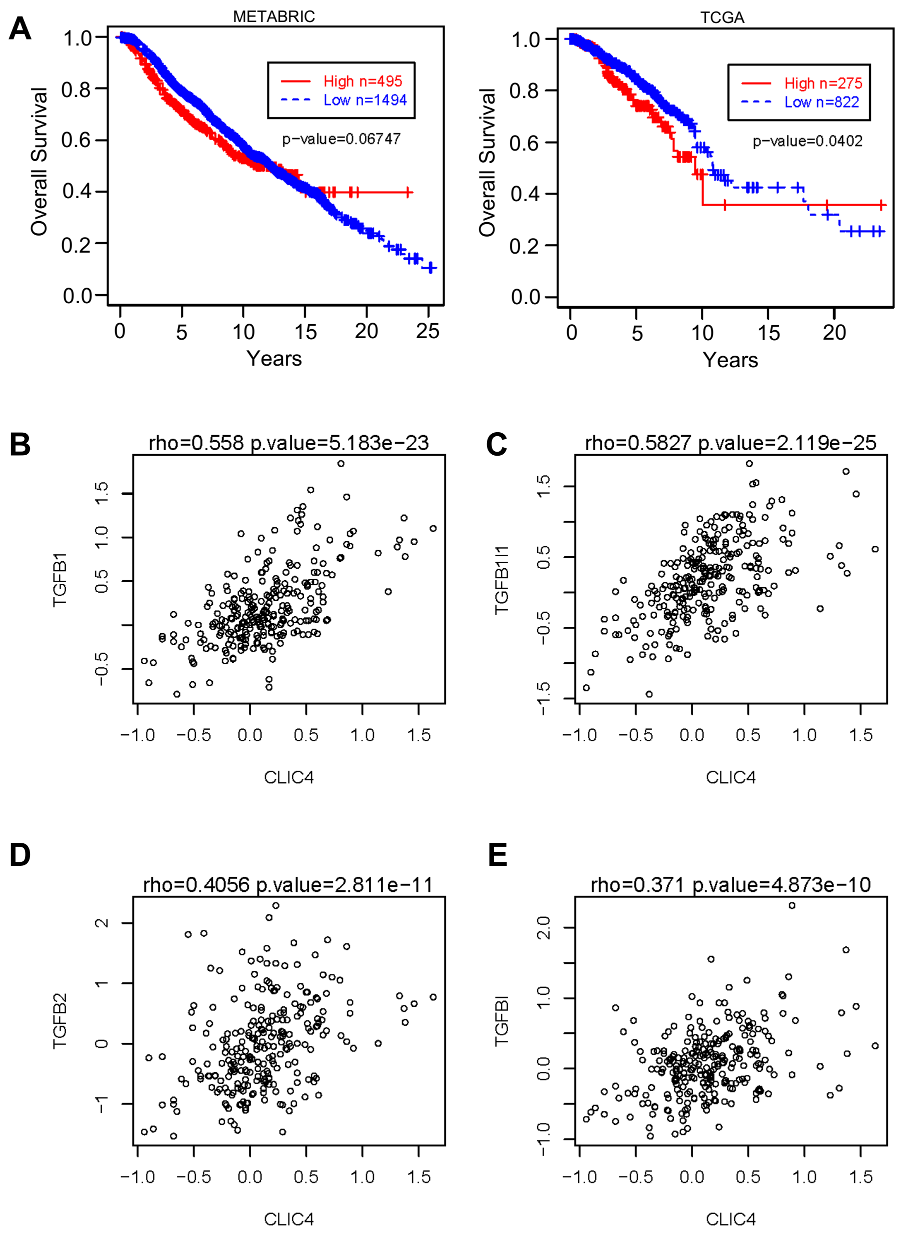

S1 Fig. Several datasets show that expression of CLIC4 and TGF-β pathway proteins are highly correlated.

A METABRIC and TCGA-BRCA breast cancer datasets, with 75th percentile as the cut-off and 25-year censored data, show the high *CLIC4* group had poor survival compared to the low *CLIC4* expression group compared by the Kaplan-Meier curves in the first 10 years. **B-E** Correlation between CLIC4 and the four most significant proteins, TGFB1 (**B**), TGFB1I1 (**C**), TGFB2 (**D**), and TGFBI (**E**) with the Spearman rho values greater than 0.37 and p-values less than 5e-10. Datasets from the TCGA Breast Cancer Proteome with n=108 cases and the Prospective Breast BI Proteome with n=125 cases were combined. The Spearman correlations between CLIC4 and eight TGF-β pathway components were calculated. Six out of the eight proteins were significant with the Spearman rho values greater than 0.17 and p-values less than 8e-3.
