# Supplementary material for: Host CLIC4 expression in the tumor microenvironment is essential for breast cancer metastatic competence
Source: PLoS Genet. 2022 Jun 21;18(6):e1010271. doi: 10.1371/journal.pgen.1010271 (PMC9249210; doi:10.1371/journal.pgen.1010271)
Supplement: S2 Fig — A Immunoblot showing CLIC4 protein expression in the 6DT1 and E0771 cells used in this study. B Primary tumor weight (g = grams) and lung metastasis quantification at 14, 21, and 28 days after implantation of 1×10^5^ 6DT1 cells into the fourth left mammary fat pad of FVB female mice. C-D Representative hematoxylin and eosin staining (C) and CLIC4 immunohistochemistry (D) of primary tumor and lung tissues at 14, 21, and 28 days. Whole tissue (left, scale bar = 3 mm) and high magnification (right, scale bar = 10 μm) images are shown. (PDF) [file pgen.1010271.s002.pdf]

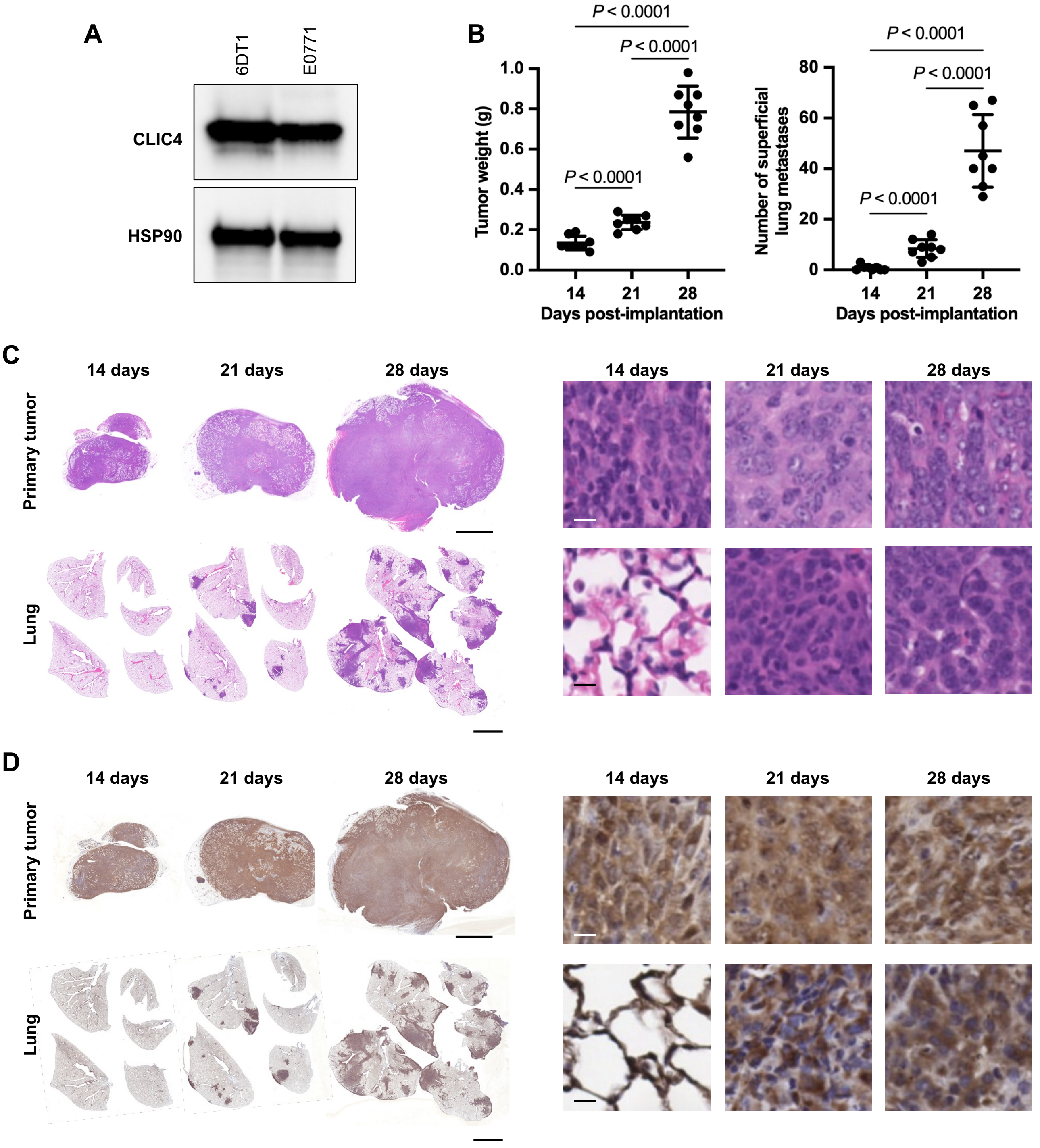

S2 Fig. Orthotopic grafting of 6DT1 cells in the mammary fat pad yields consistent primary tumor growth and reproducible lung metastases.

A Immunoblot showing CLIC4 protein expression in the 6DT1 and E0771 cells used in this study.

B Primary tumor weight (g=grams) and lung metastasis quantification at 14, 21, and 28 days after implantation of $1{\times}10^5$ 6DT1 cells into the fourth left mammary fat pad of FVB female mice.

C-D Representative hematoxylin and eosin staining (**C**) and CLIC4 immunohistochemistry (**D**) of primary tumor and lung tissues at 14, 21, and 28 days. Whole tissue (left, scale bar = 3 mm) and high magnification (right, scale bar = 10 μm) images are shown.
