# Supplementary material for: Host CLIC4 expression in the tumor microenvironment is essential for breast cancer metastatic competence
Source: PLoS Genet. 2022 Jun 21;18(6):e1010271. doi: 10.1371/journal.pgen.1010271 (PMC9249210; doi:10.1371/journal.pgen.1010271)
Supplement: S3 Fig — Top 100 most significant (FDR <0.05, fold-change >2) differentially expressed genes in 6DT1 primary tumors from Clic4 knockout (KO) hosts compared to those from wildtype (WT) hosts at 14 days after mammary gland implantation. For each gene, FPKM values were transformed using the function log2(x+1) and the z-statistic was computed across the 12 tumor samples. (PDF) [file pgen.1010271.s003.pdf]

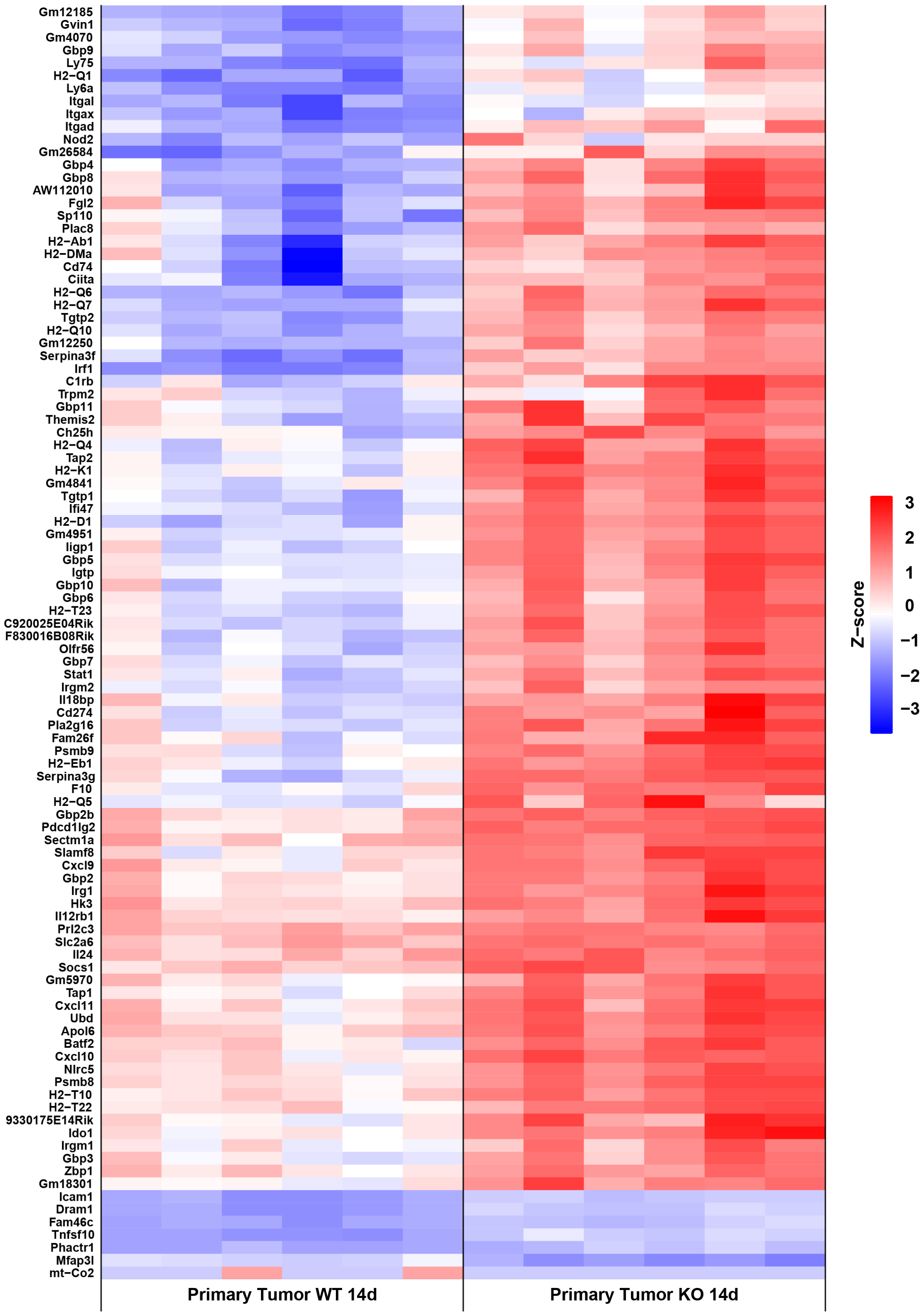

S3 Fig. RNA-seq analysis reveals numerous differentially expressed genes in primary tumors of *Clic4* knockout vs. wildtype host mice.

Top 100 most significant (FDR <0.05, fold-change >2) differentially expressed genes in 6DT1 primary tumors from *Clic4* knockout (KO) hosts compared to those from wildtype (WT) hosts at 14 days after mammary gland implantation. For each gene, FPKM values were transformed using the function log2(x+1) and the z-statistic was computed across the 12 tumor samples.
