# Supplementary material for: Host CLIC4 expression in the tumor microenvironment is essential for breast cancer metastatic competence
Source: PLoS Genet. 2022 Jun 21;18(6):e1010271. doi: 10.1371/journal.pgen.1010271 (PMC9249210; doi:10.1371/journal.pgen.1010271)
Supplement: S4 Fig — The ImmuCC signature genes (42) were compared to the genes differentially expressed (FDR <0.05, fold-change >2) between the tumors derived from Clic4 wildtype (WT) and those from knockout (KO) host mice. A Principal component analysis (PCA) show distinct separation of the tumors from Clic4 wildtype (WT) and knockout (KO) mice based on the expression of the ImmuCC signature genes. B Venn diagram depicting the overlap between the tumor differentially expressed genes (DEGs) and the ImmuCC signature genes. Forty of the 186 DEGs overlapped the ImmuCC signature, a significant overlap with a p-value of 3.5e-30. C Heat map of the 40 genes represented by the overlap in (B). D Three imputed cell fractions, monocyte, M1 macrophage, and Th17, were significantly different in the tumors of Clic4 wildtype (WT) and knockout (KO) host mice. P values were computed using the Wilcoxon rank sum test. (PDF) [file pgen.1010271.s004.pdf]

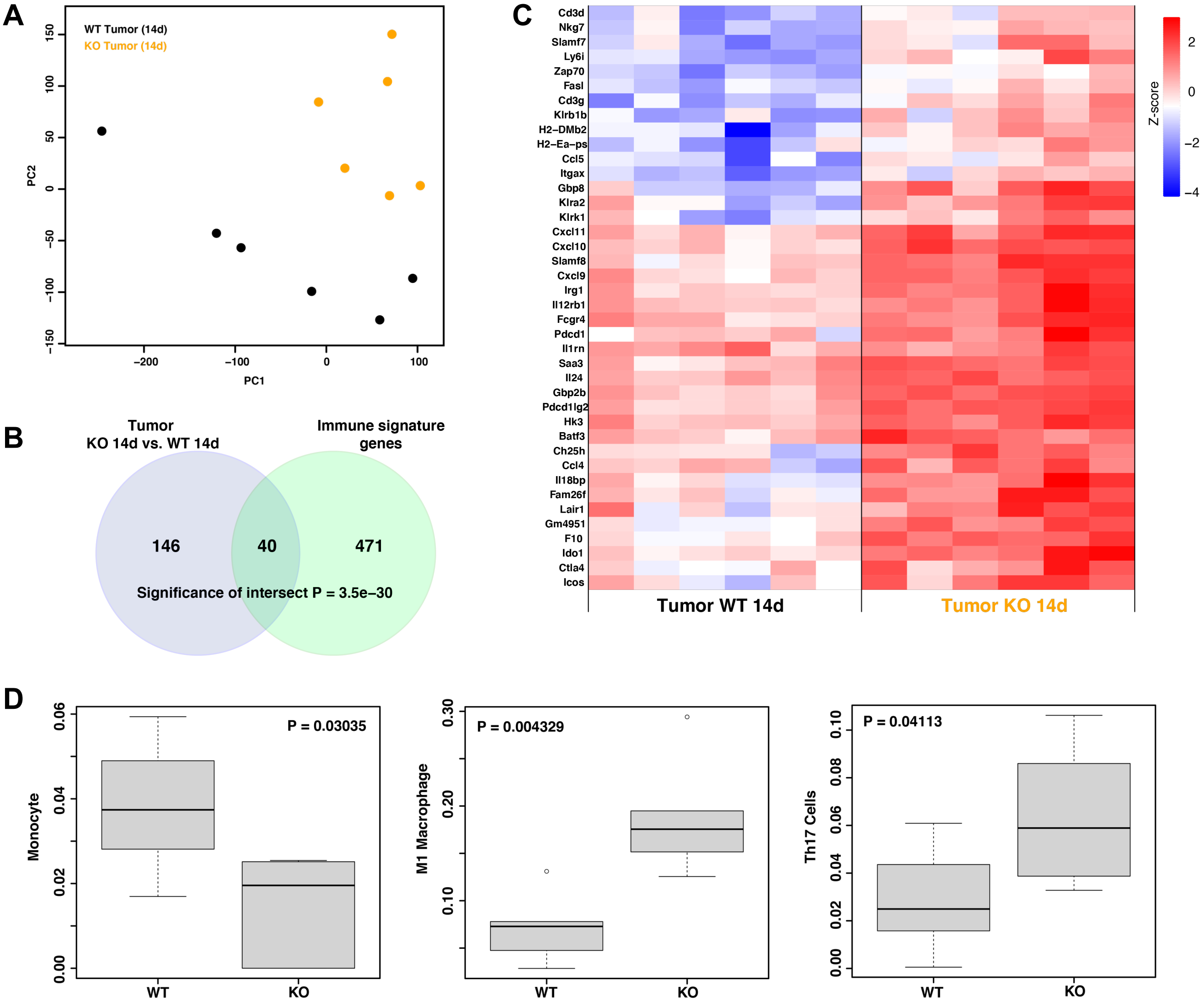

S4 Fig. Immune cell composition inferred from RNA-seq analysis suggests that immune cell differentiation or recruitment is altered in tumors of *Clic4*-deficient hosts.

The ImmuCC signature genes (42) were compared to the genes differentially expressed (FDR <0.05, fold-change >2) between the tumors derived from *Clic4* wildtype (WT) and those from knockout (KO) host mice.

A Principal component analysis (PCA) show distinct separation of the tumors from *Clic4* wildtype (WT) and knockout (KO) mice based on the expression of the ImmuCC signature genes.

B Venn diagram depicting the overlap between the tumor differentially expressed genes (DEGs) and the ImmuCC signature genes. Forty of the 186 DEGs overlapped the ImmuCC signature, a significant overlap with a p-value of 3.5e-30.

C Heat map of the 40 genes represented by the overlap in (**B**).

D Three imputed cell fractions, monocyte, M1 macrophage, and Th17, were significantly different in the tumors of *Clic4* wildtype (WT) and knockout (KO) host mice. P values were computed using the Wilcoxon rank sum test.
