# Supplementary material for: Host CLIC4 expression in the tumor microenvironment is essential for breast cancer metastatic competence
Source: PLoS Genet. 2022 Jun 21;18(6):e1010271. doi: 10.1371/journal.pgen.1010271 (PMC9249210; doi:10.1371/journal.pgen.1010271)
Supplement: S5 Fig — A mRNA expression of Clic family members by RNA-seq analysis of lungs from control and tumor-bearing Clic4 wildtype (WT) or knockout (KO) mice 14 days post-implantation of 6DT1 cells, n = 4 lungs per control group; n = 6 lungs per tumor-bearing group. Values are expressed as the FPKM mean ± s.d. B Lack of CLIC4 protein expression in Clic4 knockout (KO) animals was confirmed by Western blot of protein extracted from primary lung fibroblasts of Clic4 wildtype (WT) or KO mice. C Significantly (FDR <0.25, fold-change >1.5) differentially expressed genes in Clic4 knockout (KO) lung tissue at 14 days-post tumor implantation vs. control lung tissue. D Significantly (FDR <0.25, fold-change >1.5) differentially expressed genes in Clic4 wildtype (WT) lung tissue at 14 days-post tumor implantation vs. control lung tissue. (PDF) [file pgen.1010271.s005.pdf]

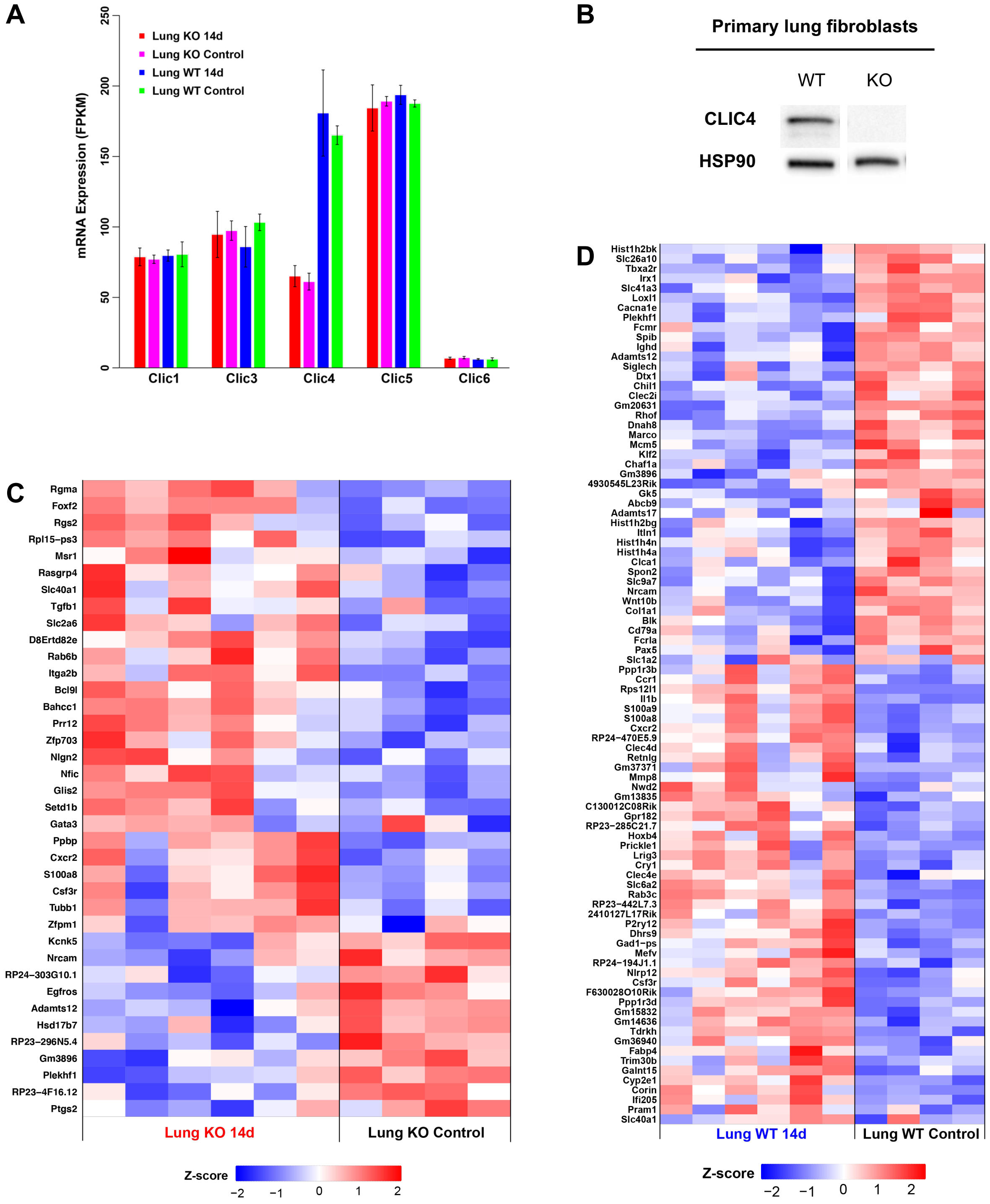

S5 Fig. RNA-seq analysis reveals unique gene expression changes in the lungs of *Clic4* knockout vs. wildtype host mice at pre-metastatic timepoints.

A mRNA expression of *Clic* family members by RNA-seq analysis of lungs from control and tumor-bearing *Clic4* wildtype (WT) or knockout (KO) mice 14 days post-implantation of 6DT1 cells, n=4 lungs per control group; n=6 lungs per tumor-bearing group. Values are expressed as the FPKM mean ± s.d.

B Lack of CLIC4 protein expression in *Clic4* knockout (KO) animals was confirmed by Western blot of protein extracted from primary lung fibroblasts of *Clic4* wildtype (WT) or KO mice.

C Significantly (FDR <0.25, fold-change >1.5) differentially expressed genes in *Clic4* knockout (KO) lung tissue at 14 days-post tumor implantation vs. control lung tissue.

D Significantly (FDR <0.25, fold-change >1.5) differentially expressed genes in *Clic4* wildtype (WT) lung tissue at 14 days-post tumor implantation vs. control lung tissue.
