# Supplementary material for: Host CLIC4 expression in the tumor microenvironment is essential for breast cancer metastatic competence
Source: PLoS Genet. 2022 Jun 21;18(6):e1010271. doi: 10.1371/journal.pgen.1010271 (PMC9249210; doi:10.1371/journal.pgen.1010271)
Supplement: S6 Fig — Broad summary of genotype-dependent differences in the primary tumor, circulation, and lungs of Clic4 wildtype (WT) and knockout (KO) mice at 14 and 28 days after tumor cell implantation. Based on proteomic and transcriptional data, primary tumors growing in a CLIC4-deficient microenvironment display enhanced necrosis, reduced angiogenesis, higher reactive oxygen species (ROS), and a more active inflammatory milieu (↑ M1 macrophages, INFg, IL6, STAT3, CCL3, CCL5). Differences in plasma circulating factors are also apparent at 14 days. Genotype-dependent differences in the lung milieu at 14 days are similar to those detected in the primary tumor. Lung inflammation and ROS are increased, while angiogenesis and TGF-β pathway signaling are reduced. These differences reflect a common host response to CLIC4 absence in the presence of a primary tumor, but which are responsible for the large difference in metastatic competence has yet to be determined. (Created with BioRender.com). (PDF) [file pgen.1010271.s006.pdf]

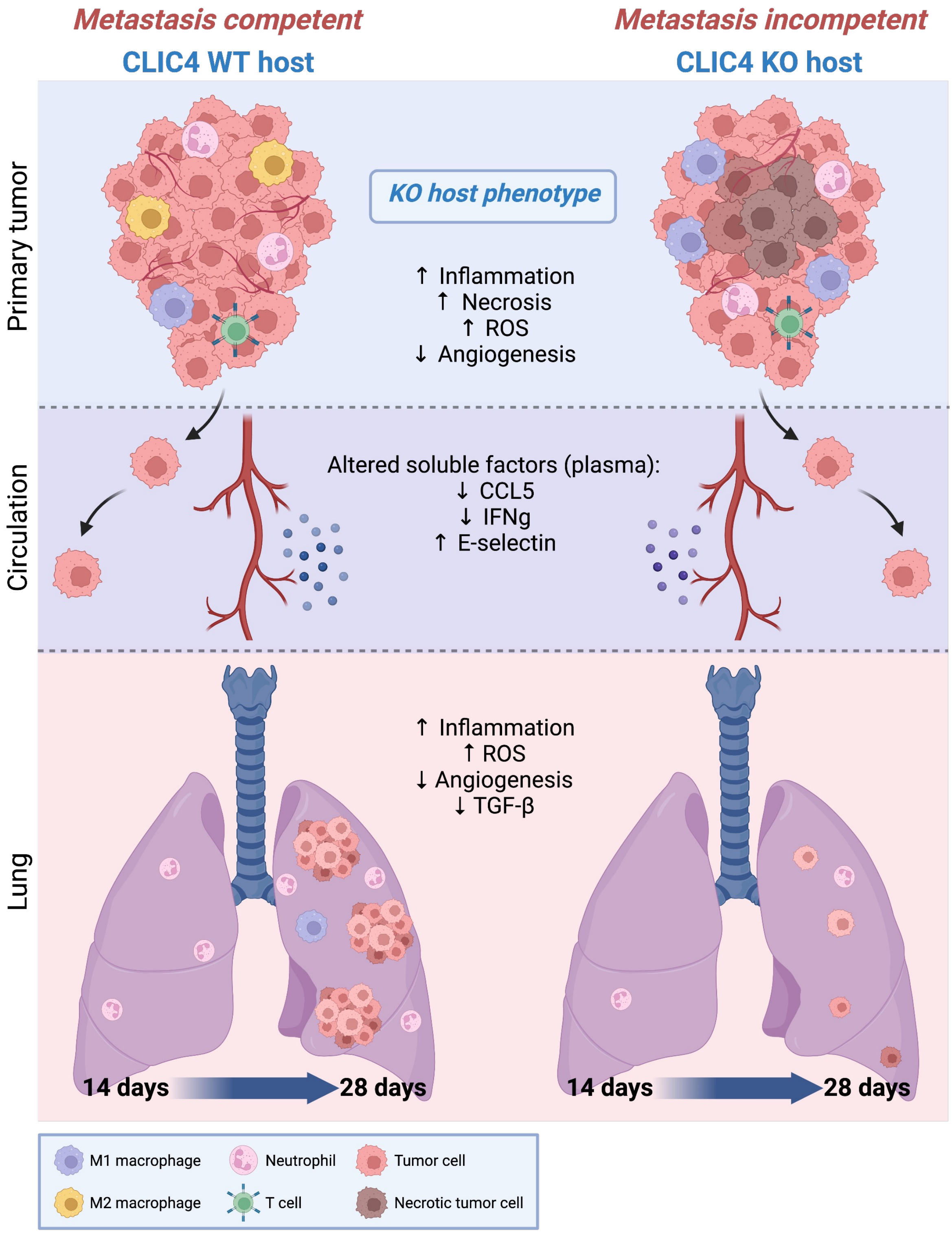

S6 Fig. Deletion of CLIC4 from the mouse genome prevents lung metastasis of implanted breast cancer cells. Broad summary of genotype-dependent differences in the primary tumor, circulation, and lungs of CLIC4 wildtype (WT) and knockout (KO) mice at 14 and 28 days after tumor cell implantation. Based on proteomic and transcriptional data, primary tumors growing in a CLIC4-deficient microenvironment display enhanced necrosis, reduced angiogenesis, higher reactive oxygen species (ROS), and a more active inflammatory milieu (↑ M1 macrophages, INFg, IL6, STAT3, CCL3, CCL5). Differences in plasma circulating factors are also apparent at 14 days. Genotype-dependent differences in the lung milieu at 14 days are similar to those detected in the primary tumor. Lung inflammation and ROS are increased, while angiogenesis and TGF-β pathway signaling are reduced. These differences reflect a common host response to CLIC4 absence in the presence of a primary tumor, but which are responsible for the large difference in metastatic competence has yet to be determined. (Created with BioRender.com)
